# NEK4 kinase regulates EMT to promote lung cancer metastasis

**DOI:** 10.1111/jcmm.13857

**Published:** 2018-09-24

**Authors:** Nian‐Hua Ding, Lu Zhang, Zhi Xiao, Zhuo‐Xian Rong, Zhi Li, Jiang He, Lin Chen, Dan‐Min Ou, Wei‐Hua Liao, Lun‐Quan Sun

**Affiliations:** ^1^ Center for Molecular Medicine Xiangya Hospital Changsha China; ^2^ Key laboratory of Molecular Radiation Oncology Hunan Province Changsha China; ^3^ Department of Radiology Xiangya Hospital Changsha China; ^4^ Deparment of Breast Cancer Medicine Xiangya Hospital Central South University Changsha China

**Keywords:** EMT, invasion, lung cancer, migration, NEK4

## Abstract

Epithelial‐to‐mesenchymal transition (EMT) is a dynamic transitional state from the epithelial to mesenchymal phenotypes. Numerous studies have suggested that EMT and its intermediate states play important roles in tumor invasion and metastasis. To identify novel regulatory molecules of EMT, we screened a siRNA library targeting human 720 kinases in A549 lung adenocarcinoma cells harboring E‐cadherin promoter‐luciferase reporter vectors. NIMA‐related kinase‐4 (NEK4) was identified and characterized as a positive regulator of EMT in the screening. Suppression of NEK4 resulted in the inhibition of cell migration and invasion, accompanying with an increased expression of cell adhesion‐related proteins such as E‐cadherin and ZO1. Furthermore, NEK4 knockdown caused the decreased expression of the transcriptional factor Zeb1 and Smads proteins, which are known to play key roles in EMT regulation. Consistently, overexpression of NEK4 resulted in the decreased expression of E‐cadherin and increased expression of Smad3. Using a mouse model with tail vein injection of NEK4 knockdown stable cell line, we found a lower rate of tumor formation and metastasis of the NEK4‐knockdown cells in vivo. Thus, this study demonstrates NEK4 as a novel kinase involved in regulation of EMT and suggests that NEK4 may be further explored as a potential therapeutic target for lung cancer metastasis.

## 1 INTRODUCTION

The epithelial‐to‐mesenchymal transition (EMT) is a cellular process during which cells lose their epithelial phenotype and acquire mesenchymal characteristics. The important role of EMT and its intermediate states have been extended to cancer cell migration and invasion.^1^ Cell plasticity owned in the process of EMT is associated with cancer stem cell‐like features and increased resistance to both radiotherapy and chemotherapy.^2^, ^3^, ^4^ During the EMT process, the adherens and tight junction proteins E‐cadherin and ZO1 lost their expression, accompanied with the increased expression of the mesenchymal proteins, such as N‐cadherin, fibronectin and vimentin. The extracellular domain of E‐cadherin forms the homologous dimers between neighboring cells and links cell–cell adherens junction(AJ).^5^ The cytoplasmic tails of ZO proteins attach to actin filaments, thus contributing to the strength and integrity of tight junctions.^6^ Following a series changes during EMT, epithelial cells lose cell‐cell contacts and apical‐basal polarity while allowing the cells for enhanced ability of invasion and metastasis.^7^ Upon transition to a mesenchymal state, cancer cells form distant metastasis through the blood or lymphatic system. In view of these findings, multiple strategies have been designed to interfere with EMT to identify potential therapeutic targets for cancer treatment, by reversing EMT to MET phenotype and reducing the drug‐resistance.^8^

Studies have suggested that EMT is a complexed and well‐coordinated process, in which the extracellular signal molecules, transcription factors, miRNAs, epigenetic and posttranslational regulation all play critical roles.^9^, ^10^ The transforming growth factor‐β (TGF‐β) as an EMT‐induction factor induces EMT through a variety of signal pathways as well as the classical TGFβ‐Smad pathway. Activated Smads combine with Smad4 to regulate the transcription of many TGF‐β/Smad target genes such as E‐cadherin. Smurf1 and Smurf2 target the activated Smads for polyubiquitylation and degradation.^11^ Other transcription factors also play a role in EMT, such as Snail, Twist and ZEBs.^12^, ^13^ ZEB expression is induced in response to TGF‐β and the induction of ZEB expression is post‐transcriptionally repressed by microRNAs (miRNAs).^14^ Zeb1 could bind to the E‐box elements of E‐cadherin promoter and suppress the expression of E‐cadherin.^15^ However, how the multi‐level regulation of EMT in epithelial cells is coordinated remains largely unknown.

In the present study, we aimed at identification of new protein kinases in EMT regulation using the human kinase siRNA library screening in lung cancer cells harboring E‐cadherin promoter‐reporter construct. We found that the Never in Mitosis A (NIMA)‐related kinase‐4 (NEK4) was a positive regulator for lung cancer EMT, resulting in an increased potential for cells to migrate and invade.

## 2 MATERIALS AND METHODS

### 2.1 Cell culture

Human lung carcinoma cell line A549 cells and human breast adenocarcinoma MDA‐MB‐231 cells were obtained from the Cancer Research Institute of Central South University. Cell line MCF7 cells were obtained from the Cell Bank of the Chinese Academy of Sciences (Shanghai, China). A549 cells were cultured in RPMI 1640 medium (Gibco, China) supplemented with 10% fetal bovine serum (Gibco, New York, USA) and 1% penicillin/streptomycin (Invitrogen, New York, USA). MDA‐MB‐231 cells were cultured in DMEM medium (Gibco) supplemented with 10% fetal bovine serum and 1% penicillin/streptomycin. MCF7 cells were cultured in DMEM/F12 (1:1) medium supplemented with 10% fetal bovine serum and 1% penicillin/streptomycin. Cell cultures were grown in a humidified incubator at 37°C and 5% CO_2_.

### 2.2 DNA constructs

To construct E‐cadherin promoter‐luciferase reporter vector pGL‐E‐cadherin, we designed the E‐cadherin promoter sequence from −1007 to +29 of the transcriptional start point with Kpn1 and Sac1 enzyme sites. The primer sequences were shown in Table S1. The E‐cadherin promoter fragment was obtained by PCR on genomic DNA from the human A549 cell line. The PCR product was cloned into the pGL4.17[luc2/Neo] vector. The E‐cadherin promoter fragment was inserted in the upstream of the luc2 gene to regulate its transcription. The control vector pRL‐TK harboring Renilla luciferase is purchased from Promega. To establish the NEK4 overexpression system, we constructed pc‐NEK4 plasmid. The NEK4 fragment was obtained by PCR on cDNA generated by RT‐PCR of the human A549 mRNA. We designed the NEK4 sequence with HindIII and XhoI enzyme sites. The primer sequences were shown in Table S1. The PCR product was cloned into the pc‐DNA3.1+ vector.

### 2.3 NEK4 knockdown or overexpression stable cell lines

To knock down NEK4, the human shRNA NEK4 was cloned into Lenti‐X^™^ shRNA Expression Systems to generate NEK4‐shRNA. The shNEK4 sequences were shown in Table S1. In brief, 293T cells were co‐transfected with the lentiviral vectors, packaging plasmid, and envelope plasmid. The cell culture supernatant was collected 24 h after transfection and centrifuged to remove the cell debris. A549 cells were transduced with shNEK4 lentiviral particles and a negative control shCon for 48 hours and then selected with 2 μg/mL puromycin (Sigma, St Louis, MO, USA) for 1 week.

To construct the NEK4 overexpression stable cell line, A549 cells were transfected with pc‐NEK4 plasmid and a negative control pc‐DNA3.1+ for 48 hours and then selected with 500 μg/mL G418 (Sigma) for 2 weeks.

### 2.4 High‐throughput siRNA screening against human kinases

The siGENOME Smart Pool siRNA library targeted against 720 human kinases was from Dharmacon (GE, USA), with a mixture of four siRNAs targeting one kinase. Non‐silencing control siRNA was obtained from Dharmacon. For the high throughput screening, A549 cells were plated onto clear‐bottom white 96‐well plates (Corning, New York, USA) 1 day before treatment. Next day, we replaced the culture medium with fresh 1640 contained 10% FBS and 2 μg/mL TGF‐β1 (R&D, Minneapolis, MN, USA). Four hours later, A549 cells were co‐transfected with siRNA pools, pGL‐E‐cadherin plasmid and pRL‐TK plasmid with DharmaFECT Transfection Reagent (GE Dharmacon, Lafayette, CO, USA) and Fugene HD reagent (Promega, Madison, USA) according to the manufacturer's protocol. Cells were incubated for 48 hours and measured the activity of Firefly and Renilla luciferases using Dual‐Glo Luciferase Assay kit (Promega). Relative firefly luciferase activity, as the relative light unit (RLU), was normalized to Renilla luciferase activity encoded by the co‐transfected pRL‐TK plasmid. Fold change of the RLU was scored comparing with cells transfected with control siRNA. The triplicate wells were set up for each screening. The candidates in the screening reached a score >1.6 or <0.7 in two independent screens were selected. New siRNAs were synthesized from RiboBio (Guangzhou, China) for verification of the selected candidates, consistent with the Smart Pool siRNA library sequences. siRNA transfection was all operated with a mixture of four siRNAs targeting one gene.

### 2.5 RNA extraction and real‐time quantitative PCR

Total RNA was isolated using TRIZOL buffer following the manufacturer's protocol. The extracted RNA samples were quantified and reverse transcribed to cDNA with Fermentas RevertAid^™^ First Strand cDNA Synthesis Kit. Quantitative real‐time PCR was then performed using the Real‐Time PCR Detection System (Bio‐RAD, CFX96TM) according to the manufacturers’ instructions. The primer sequences were shown in Table S1. For miRNA detection, the primers were synthesized for reverse transcription and real‐time PCR by RiboBio using stem‐loop RT‐PCR method. The primer sequences were shown in Table S1. The fold change of relative expression was calculated by the 2^‐ΔΔCt^ method and normalized against the internal standard GAPDH gene or U6.

### 2.6 Antibodies and western blot analysis

In the study, the antibody sources and dilution were as follows: anti‐NEK4 (Abcam, 1:1000 dilution), anti‐E‐cadherin (CST, 1:1000 dilution), anti‐ZO‐1 (BD, 1:1000 dilution), anti‐Vimentin (Abcam, 1:1000 dilution), anti‐N‐Cadherin (CST, 1:1000 dilution), anti‐α‐Tubulin (Santa Crus, 1:500 dilution), anti‐TCF8/ZEB1 (CST, 1:1000 dilution), anti‐Smad1/2/3/4/5 (CST kit, 1:1000 dilution), anti‐SNAIL (Abcam, 1:500 dilution). For preparing total protein samples, cells grown in culture dishes with specific treatment were harvested and lysed in RIPA lysis buffer supplemented with 1X PhosStop (Roche, Mannheim, Germany) and 1X Protease inhibitor cocktail (Roche). Cell lysates were resolved on SDS‐PAGE gels and transferred to PVDF membranes. Membranes were probed against specific primary antibodies followed by HRP conjugated secondary antibodies, and then visualized using the ECL Western Blotting System (GE Healthcare).

### 2.7 Migration assay

The effect of NEK4 on migration capacity was assessed using scratch wound assay. A549 cells were seeded into 6‐well culture plates and transfected with siNEK4 or siRNA control for 24 hours. The next day, cells were grown to complete confluence. Subsequently, 3 parallel, linear wounds were produced in each dish with a 10‐μL plastic pipette tip. The cells were then cultured with low serum medium contained 1% FBS supplemented with or without TGF‐β. Cell migration was then observed by microscope at different time points. The wound area was measured using ImageJ software.

### 2.8 Transwell assay

For measuring the effect of NEK4 on invasion capacity, transwell invasion assay was performed. A549 cells were transfected with siNEK4 or siRNA control for 24 hours. Then the cells were suspended in serum‐free medium and added to the top chamber of the 24‐well, matrigel‐coated transwell chamber system (Corning). Media with 10% serum was added in the bottom chamber. Cells were incubated for 48 or 72 hours at 37°C before test. And then the membrane was fixed using 4% paraformaldehyde and stained with 0.5% crystal violet. At the end of the assay, cells on the membrane were removed using a cotton swab and the cells below the membrane were observed using a microscope.

### 2.9 Immunofluorescence

A549 cells were plated on glass cover‐slips in 6 well plates and transfected with siRNAs for 48 hours and processed for immunofluorescence. Cells on cover‐slips were washed with PBS and fixed in 4% paraformaldehyde (15 min at room temperature). Cover‐slips were re‐washed 3 times in PBS for 5 minutes and blocked by 5% BSA in PBS for 1 hour. Antibody was added in PBS containing 0.5% BSA and incubated for 16 hours at 4°C in humidified chamber. Cover‐slips were washed 3 times in PBS and incubated with fluorescent secondary antibody (Abcam, 1:1000) for 1 hour at room temperature. After washing, cover slips were mounted in DAPI, dried and stored at −20°C until microscopy. Micrographs were taken using Olympus fluorescent microscope.

### 2.10 Pulmonary metastasis model

The animal experiments were approved by the Animal Ethics Committee of Xiangya Hospital Central South University and followed the Guidelines of Animal Handling and Care in Medical Research of Hunan Province, China. To establish the human pulmonary metastasis model, 5 week‐old female nude mice were purchased from Vital River Laboratories (the original breeding pairs were purchased from Charles River). The mice were injected with 2 × 10^5^ A549‐shNEK4 or A549‐shcon cells resuspended in 100 μL of RPMI1640 medium via tail vein. 1 month later, mice were sacrificed. Lung tissues were excised, fixed with bouins fixative for 30 minutes and washed with 70% alcohol until the yellow color faded. The tumor colony number was counted under stereoscopic microscope (Leica Microsystems).

### 2.11 Hematoxylin & Eosin stain and Immunohistochemistry

For histological examination, the pulmonary tissues were fixed in 10% neutral‐buffered formalin and embedded in paraffin, and 4 μm sections were prepared for H&E stain and immunohistological assay. E‐cadherin was detected using an anti‐E‐cadherin antibody. NEK4 was detected using an anti‐NEK4 antibody.

### 2.12 Statistical analysis

Statistical analyses were performed using GraphPad prism 6.01 software. The data are presented as the means ± SD. The statistical analyses were performed using Student's *t* test or spearman. The *P *< 0.05 were considered to be statistically significant.

## 3 RESULTS

### 3.1 Identification of NEK4 protein kinase as regulator of E‐cadherin in A549 cells

**Figure 1:**
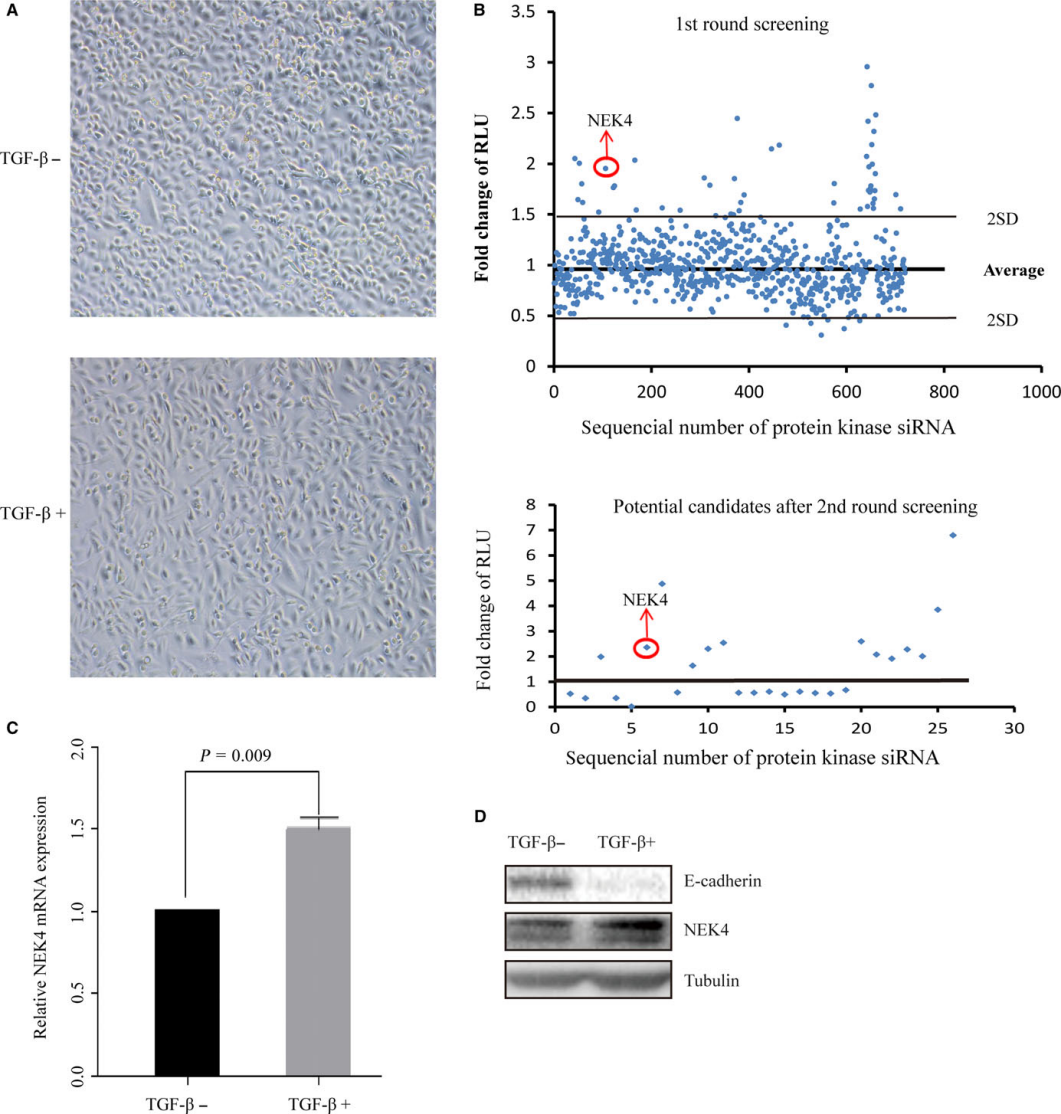
Identification of NEK4 protein kinase as regulator of E‐cadherin in A549 cells. (A) EMT model induced by TGF‐β. A549 cells were treated with TGF‐β (2 μg/mL) for 48 hours and taken for photographs under optical microscope. (B) (up panel) High‐throughput siRNA screening against human kinases. The human 720 protein kinase siRNAs were screened using A549 cell line. For each siRNA, triplet wells were set up. (lower panel) 26 potential candidates after second‐round selection. Fold change values for each siRNA were plotted to identify hits with a score >1.6 or <0.7 in two rounds screening. (C) Real‐time PCR to detect the NEK4 mRNA. A549 cells treated with or without TGF‐β (2 μg/mL, 48 hours). Data are representative of 3 independent experiments. (D)Western blots (WB) were performed to detect the NEK4 protein level in A549 cells induced with TGF‐β (2 μg/mL) or not for 48 hours

To identify potential kinases that are involved in regulation of EMT progress, we utilized a TGF‐β‐induced EMT model in A549 lung adenocarcinoma cells. After TGF‐β treatment for 48 h, the cells exhibited typical EMT morphology (Figure 1A). In this model, we performed the siRNA library targeting human kinases (totally 720), according to siRNA effect on E‐cadherin promoter activity. Based on the first‐round screening, we selected the positive hits for second‐round confirmative screening, from which we selected 13 negative candidates and 13 positive candidates according to their consistency between the results from the two rounds (Table S2 and Figure 1B). As TGF‐β treatment has been a well‐accepted means to cause EMT of the cells of epithelial origin, we examined the mRNAs expression of the candidate kinases in A549 cells treated with or without TGF‐β (Figure S1A) to validate the involvement of the candidates in TGF‐β‐induced EMT. Furthermore, the protein level of E‐cadherin was assayed upon knockdown of the candidate kinases (Figure S1B and C). In accordance with the comprehensive evaluation of the above results, we selected the NEK4 for further study of its role in regulation of TGF‐β‐induced EMT, during which the NEK4 expression level of mRNA and protein was up‐regulated (Figure 1C and D).

### 3.2 The biological function of NEK4 associated with EMT

**Figure 2:**
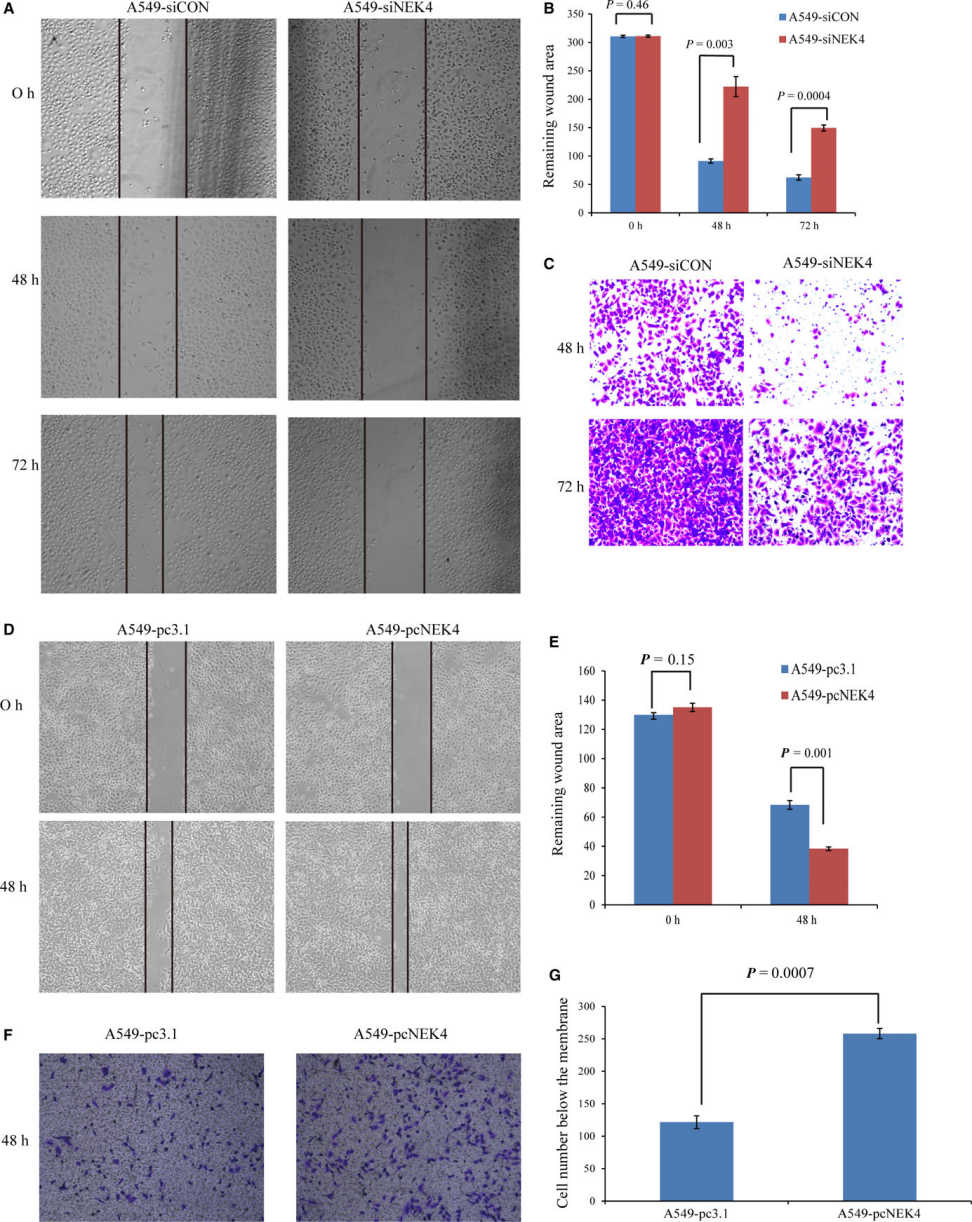
The biological function of NEK4 associated with EMT. (A) Representative images of scratch assay at different time points. A549 cells were seeded into 6‐well culture plates and transfected with siNEK4 mix or siRNA control. Cell migration was then observed by microscope at different time points. Data are representative of 3 independent experiments. (B) Graphs showed wound areas in A549 cells transfected with siNEK4 mix or siRNA control. The wound area was analyzed using ImageJ software. Bars represent mean ± SD of three measurements. (C) Representative images of the matrigel‐coated transwell assay at different time points. A549 cells were transfected with siNEK4 mix or siRNA control. Data are representative of 3 independent experiments. (D) Representative images of scratch assay at different time points. A549 cells stably transfected with plasmid pc‐DNA 3.1+ or pc‐NEK4 were seeded into 6‐well culture plates. Cell migration was then observed by microscope at different time points. Data are representative of 3 independent experiments. (E) Graphs showed remaining wound areas in A549 cells stably transfected with plasmid pc‐DNA 3.1+ or pc‐NEK4. The wound area was analyzed using ImageJ software. Bars represent mean ± SD of three measurements. (F) Representative images of the matrigel‐coated transwell assay. A549 cells were stably transfected with plasmid pc‐DNA 3.1+ or pc‐NEK4. Data are representative of 3 independent experiments. (G) Graphs showed the cell numbers below the membrane. A549 cells were stably transfected with plasmid pc‐DNA 3.1+ or pc‐NEK4. Bars represent mean ± SD of three measurements

Although NEK4 has been reported to play some roles in DNA repair and apoptosis,^16^, ^17^, ^18^, ^19^ little is known about its effect on cancer cell EMT, which is closely associated with the potential of cancer cell invasion and metastasis. As we observed that NEK4 knockdown induced robust increase of E‐cadherin promoter activity and NEK4 expression was up‐regulated during the EMT progress, we speculated that NEK4 might mediate cell invasion and metastasis by promoting EMT. To confirm this hypothesis, we investigated the function of NEK4 in matrigel‐coated transwell assay and scratch assay. We found that knockdown of NEK4 in A549 cells significantly inhibited cell migration and invasion (Figure 2A‐C). We also found a reduced migratory ability of the cells when treated with siNEK4 in the EMT model of A549 cells induced by TGF‐β (Figure S2). Furthermore, we demonstrated that over‐expression of NEK4 promoted cell migration and invasion (Figure 2D‐G). These results suggest that NEK4 is a promotor of cell invasion and migration.

### 3.3 NEK4 regulation of EMT molecular markers

**Figure 3:**
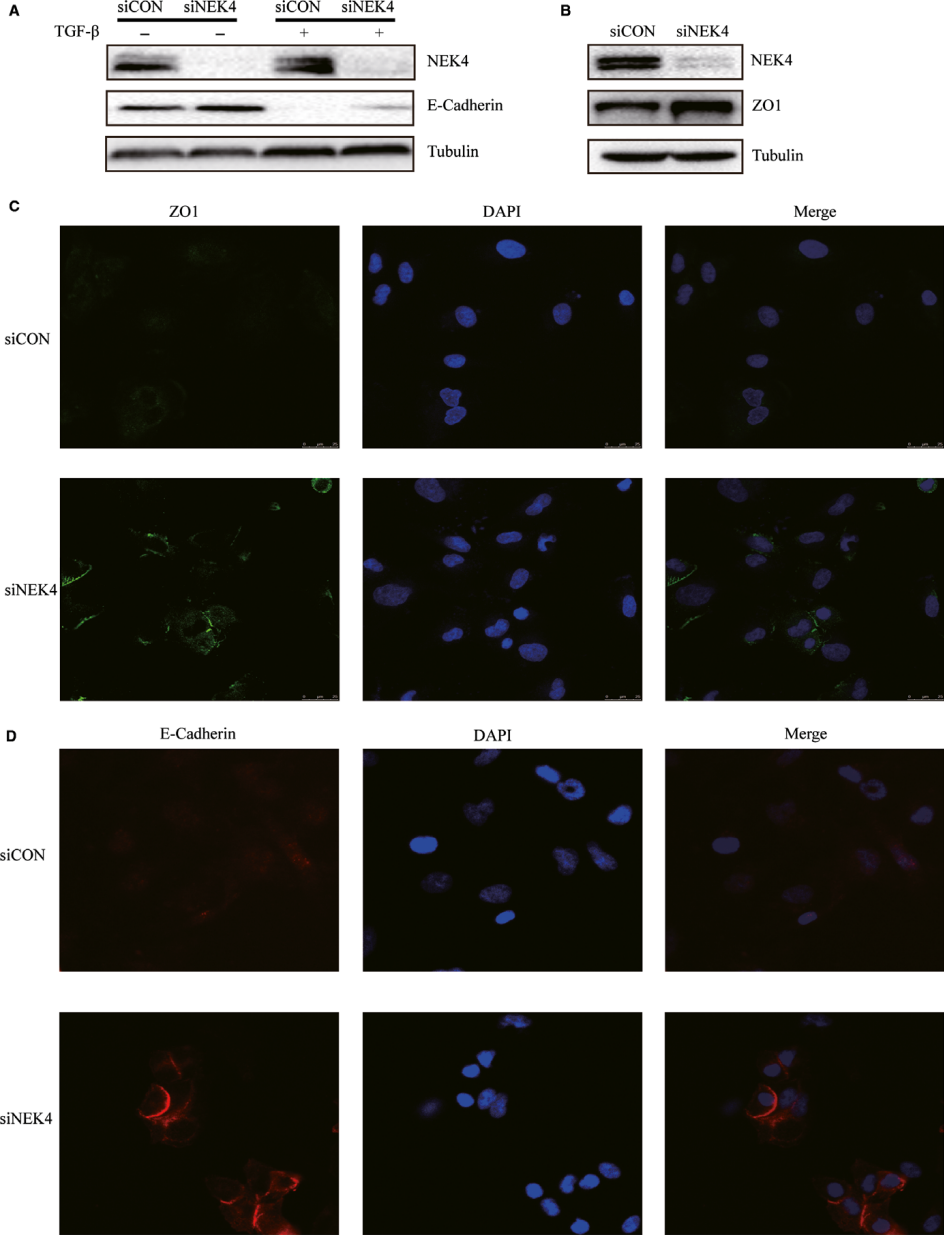
NEK4 in the regulation of EMT molecular markers. (A) Expression of E‐cadherin in A549 cells transfected with siNEK4 mix or siCon supplemented with TGF‐β (2 μg/mL) or not for 48 hours. (B) Detection of EMT marker proteins. A549 cells were transfected with siNEK4 mix or siCon for 48 hours. (C and D) Immunofluorescence technique to analyze E‐cadherin and ZO1 proteins. A549 cells were transfected with siNEK4 mix or siCon for 48 hours and the E‐cadherin and ZO1 proteins were detected by IF. Cells were observed by fluorescence microscope

The induction of EMT mostly accompanied with the decreased expression of epithelial markers such as E‐cadherin and ZO1, and the enhanced expression of mesenchymal markers such as N‐cadherin and Vimentin. To further confirm the role of NEK4 in regulation of EMT, the E‐cadherin expression was detected in A549 cells transfected with siNEK4, supplemented with TGF‐β or vehicle control. The E‐cadherin expression was up‐regulated when treated with siNEK4, in the presence or absence of TGF‐β (Figure 3A). We also examined another epithelial marker ZO1 and found that it was up‐regulated in A549 cells transfected with siNEK4 (Figure 3B). However, no obvious changes in mesenchymal markers such as N‐cadherin and Vimentin were observed when NEK4 was knockdown (Figure S3), indicating that NEK4 may only be involved in the loss of epithelial phenotype, but not in acquisition of mesenchymal features. Immunofluorescence staining further confirmed that the E‐cadherin and ZO1 proteins were up‐regulated in A549 cells transfected with siNEK4 (Figure 3C and D). The above results verified that the EMT‐associated epithelial markers such as E‐cadherin and ZO1 could be up‐regulated in A549 cells transfected with siNEK4.

### 3.4 NEK4 regulation of EMT‐associated transcription factors and signal transduction

**Figure 4:**
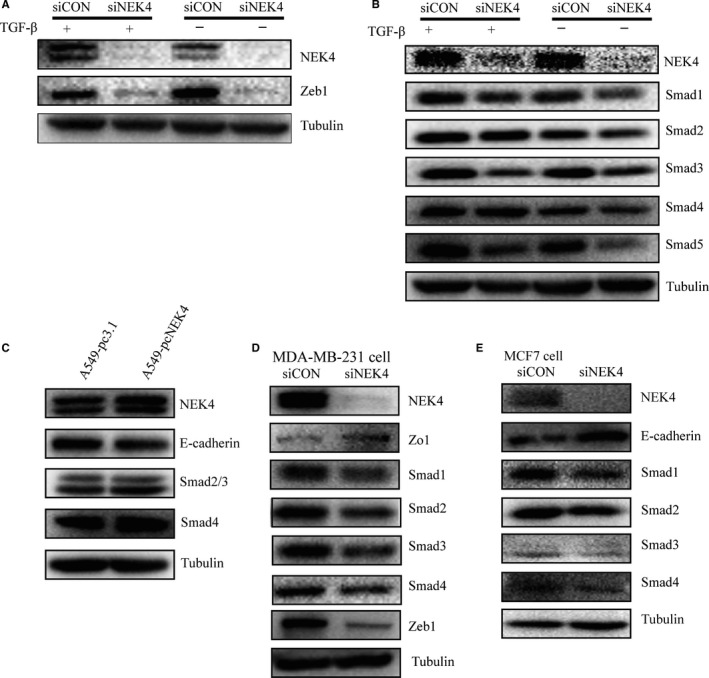
NEK4 regulation of EMT associated transcription factors and signal transduction. (A) WB to detect the EMT transcription factors Zeb1. A549 cells were transfected with siNEK4 mix or siCon supplemented with TGF‐β (2 μg/mL) or not for 48 hours. (B) WB to detect the Smads proteins in A549 cells transfected with siNEK4 mix or siCon and supplement with TGF‐β (2 μg/mL) or not for 48 hours. (C) WB to detect the proteins in A549 cells stably transfected with plasmid pc‐DNA 3.1+ or pc‐NEK4. (D) WB to detect the proteins in MDA‐MB‐231 cells transfected with siNEK4 mix or siCon for 48 hours. (E) WB to detect the proteins in MCF7 cells transfected with siNEK4 mix or siCon for 48 hours

As NEK4 was selected based on its impact on E‐cadherin promotor activity upon TGF‐β treatment, we next examined the effect of NEK4 on the EMT‐related transcription factors. Snail and ZEB transcriptional regulators have emerged as key regulatory nodes for EMT.^16^ In A549 cells, we found that when NEK4 was inhibited, the expression of ZEB1 was decreased while Snail1 was not affected (Figure 4A, Figure S4A), implying that NEK4‐mediated EMT may be via ZEB1.

In the canonical TGF‐β pathway, signaling to the transcriptional machinery is transduced by a unique family of intracellular signaling mediators, called Smads. To explore if NEK4 is involved in TGF‐β signal transduction, we further tested the effect of knockdown of NEK4 on Smads. As shown in Figure 4B, among Smad 1 to Smad 5, Smad3 was most affected molecule upon NEK4 inhibition. This supports the notion that NEK4 could be a critical regulator for EMT as Smad3 has been suggested to be one of the key signal transducer of TGF‐β pathway. We also confirmed the down‐regulation of E‐cadherin and the up‐regulation of Smad3 in NEK4 overexpression stable cell line A549‐pcNEK4 (Figure 4C). In order to exclude the possibility that the observed effect of NEK4 was cell‐ and cancer‐type specific, we employed two more breast cancer cell lines, MDA‐MB‐231 and MCF7, to validate molecular effect of NEK4. We found that knockdown of NEK4 in MDA‐MB‐231 and MCF7 resulted in up‐regulation of E‐cadherin or ZO1, and down‐regulation of ZEB1 and Smads (Figure 4D and E), which was consistent with that in A549 cells.

Considering miR200 family has been suggested to be important regulators of EMT,^14^, ^17^ we also examined the effect of NEK4 on miR200 levels and found no significant changes in A549 cells with NEK4 knockdown (Figure S4B). Together, the present data implied that the NEK4 regulated the TGF‐β‐induced EMT through Smads and ZEB1.

### 3.5 NEK4 promotion of invasion and migration in vivo

**Figure 5:**
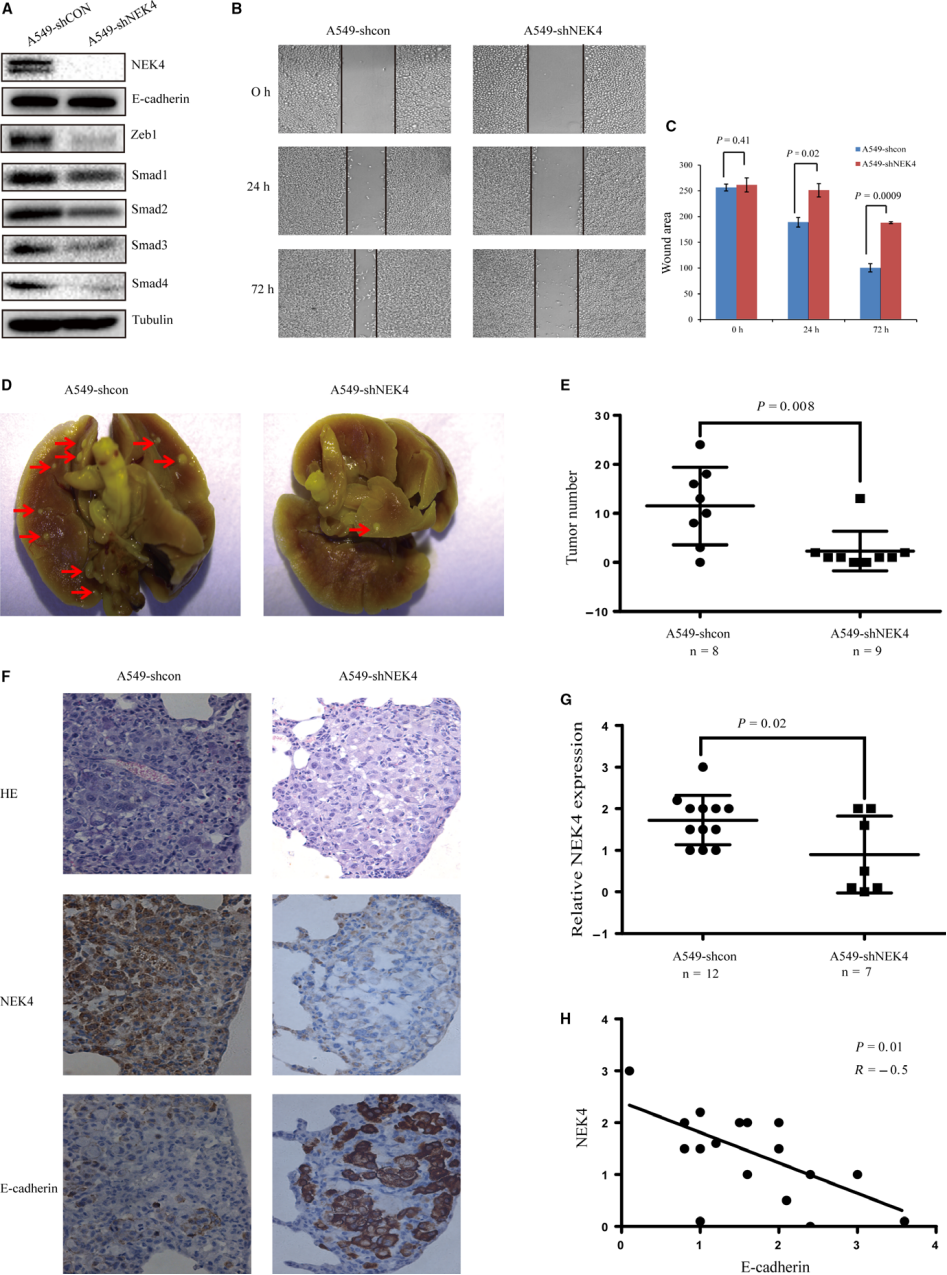
NEK4 promotion of invasion and migration in vivo. (A) WB to detect the proteins in A549 cells stably transfected with shNEK4 or shCon. (B) Representative images of scratch assay at different time points. A549 cells were stably transfected with shNEK4 or shCon. (C) Graphs showed remaining wound areas in A549 cells stably transfected with shNEK4 or shCon at different time points. Bars represent mean ± SD of three measurements. Data are representative of 3 independent experiments. (D) Lung tumor clone formation in mouse injected with A549‐shcon or A549‐shNEK4 cells for 1 month. The whole lung specimens were fixed with bouins and observed with stereoscopic microscope. (E) Statistical analysis of tumor clone number. Tumor clones were counted in the whole lung and analyzed between A549‐shcon and A549‐shNEK4 group. (F) HE stain and IHC to evaluate the pathological features. Tissue slides were stained with HE and observed using a microscope to detect every tumor clone in the whole lung. The expression of NEK4 and E‐cadherin in lung tissue slides were detected with IHC. (G) Statistical analysis of the relative NEK4 expression in two groups. The score was given as a sum of each stain intensity multiplied by the percentage of stained cells in every tumor clone. (H) Correlation analysis between the expression of NEK4 and E‐cadherin. The score was given as a sum of each stain intensity multiplied by the percentage of stained cells in every tumor clone

To further verify the function of NEK4 in vivo, we established stable A549‐shNEK4 cells. The NEK4 knockdown efficiency was tested, confirming up‐regulation of E‐cadherin and inhibition of ZEB1 and Smads in A549‐shNEK4 cells (Figure 5A). Also cell migration ability was reduced in A549‐shNEK4 cells (Figure 5B and C). These results were consistent with the alterations detected in A549 cells transfected with siNEK4. For testing in vivo function of NEK4, we established a pulmonary metastasis mouse model with the tail vein injection of A549‐shNEK4 or A549‐shcon cells to investigate whether the knockdown of NEK4 impacted tumor invasion and metastasis in vivo. One month after the injection of A549 cells, mice were sacrificed and pulmonary excised and fixed to count the colony number in lung. The colony number of the pulmonary tumors formed in the mice with A549‐shNEK4 cells injection was markedly lower than that in the mice with A549‐shcon cells, indicating that NEK4 contributed to tumor invasion and metastasis (Figure 5D and E). For histological examination, the mouse lung tissue slides were examined for expression of NEK4 and E‐cadherin by IHC. The score was given as a sum of staining intensity multiplied by the percentage of stained cells in every tumor clone. IHC results confirmed a decreased expression of NEK4 in the tumor of A549‐shNEK4 group, and this verified the knockdown effects of shNEK4 (Figure 5F and G). Statistical analysis showed a negative correlation between NEK4 and E‐cadherin levels, which showed that the decreased NEK4 expression accompanied with the E‐cadherin up‐regulation (Figure 5F and H). Hence, these results indicated that the knockdown of NEK4 increased the expression of E‐cadherin and inhibited the tumor formation in the mice pulmonary.

## 4 DISCUSSION

EMT and its intermediate states play important role in tumor invasion and metastasis. The cell plasticity displayed in the EMT is associated with cancer stem cell‐like features and increased resistance to chemotherapy.^4^, ^5^ Multiple strategies to interfere with EMT have been proposed, aiming to identify new druggable targets in the EMT process or revert mesenchymal cells back to an epithelial state to improve the drug sensitivity.^8^ Thus, uncovering complexed mechanisms of EMT would potentially provide novel strategy to clinically prevent metastases and the outgrowth of therapy‐resistant cancer stem cells in cancer patients. In present study, we identified NEK4 through siRNA library screening as a positive regulator of EMT, which not only contributed to better understanding of EMT process, but also suggest a potential therapeutic target.

NEK4 is one of the Nek family members and overexpressed in lung cancer and colorectal cancer.^18^, ^19^ Nek protein family includes 11 subtypes and belongs to serine‐threonine kinases.^20^ The Nek proteins share substantial homology at the amino terminal domain but exhibit variable homology at the carboxyl region. Nek2 and 8 have been mostly studied and participate in the regulation of cell cycle and cell polarity.^21^ They can phosphorylate the centrosome complex and accelerate mitosis.^22^ For NEK4, there have been some reports showing that NEK4 suppression caused defects in DNA repair and sensitized cancer cells to apoptosis,^23^ which suggests the roles of NEK4 in tumor progression. In this study, we found that NEK4 could regulate cell invasion and migration. When treated with siNEK4 in A549 cells, the cell invasion and migration was suppressed in normal growth situation or in the EMT progress induced by TGF‐β. The overexpression of NEK4 promoted the cell invasion and migration. The biological significance was emphasized by the up‐regulation of E‐cadherin expression in A549 cells treated with siNEK4, cultured in normal medium or supplemented with TGF‐β. The E‐cadherin was also down‐regulated in A549 cells stably transfected with plasmid pc‐NEK4. In the meantime, we detected the up‐regulation of epithelial marker ZO1 and no obvious changes of the mesenchymal markers such as Vimentin and N‐cadherin in A549 cells transfected with siNEK4. ZO1 is an epithelial marker and the diffusion of ZO1 from cell‐cell contacts results in the dissolution of tight junctions during EMT.^24^ We propose that NEK4 may mainly contribute to the process of loss of epithelial characteristics.

The EMT progression is regulated at multiple levels, including receptor mediated signal transduction, transcription activation and epigenetic regulation such as miRNAs. Our screening results indicated that NEK4 knockdown led to a higher luciferase activity. This implied that NEK4 could influence the E‐cadherin promoter activity either directly or indirectly. By examining two most important EMT transcription factors Snails and ZEBs, we found ZEB1 was the key molecule involved in NEK4‐related E‐cadherin promoter activity regulation and not regulated by miR200. Further analyzing the involved signal transducers of TGF‐β pathways, Smad3 was found to be the most significant effector molecule, although the direct substrates for NEK4 were not identified in the present study. Accordingly, NEK4 may be an important regulator during early phase of EMT via transcriptional activation of an array of EMT‐related genes.

In summary, we provided the evidence showing that NEK4 can promote EMT both in vitro and in vivo. Thus, further characterization of NEK4 both biochemically and molecularly in EMT process is warranted.

## CONFLICTS OF INTEREST

The authors confirm that there are no conflicts of interest.
