# Supplementary figures and images for: NEK4 kinase regulates EMT to promote lung cancer metastasis
Source: J Cell Mol Med. 2018 Sep 24;22(12):5877–87. doi: 10.1111/jcmm.13857 (PMC6237562; doi:10.1111/jcmm.13857)

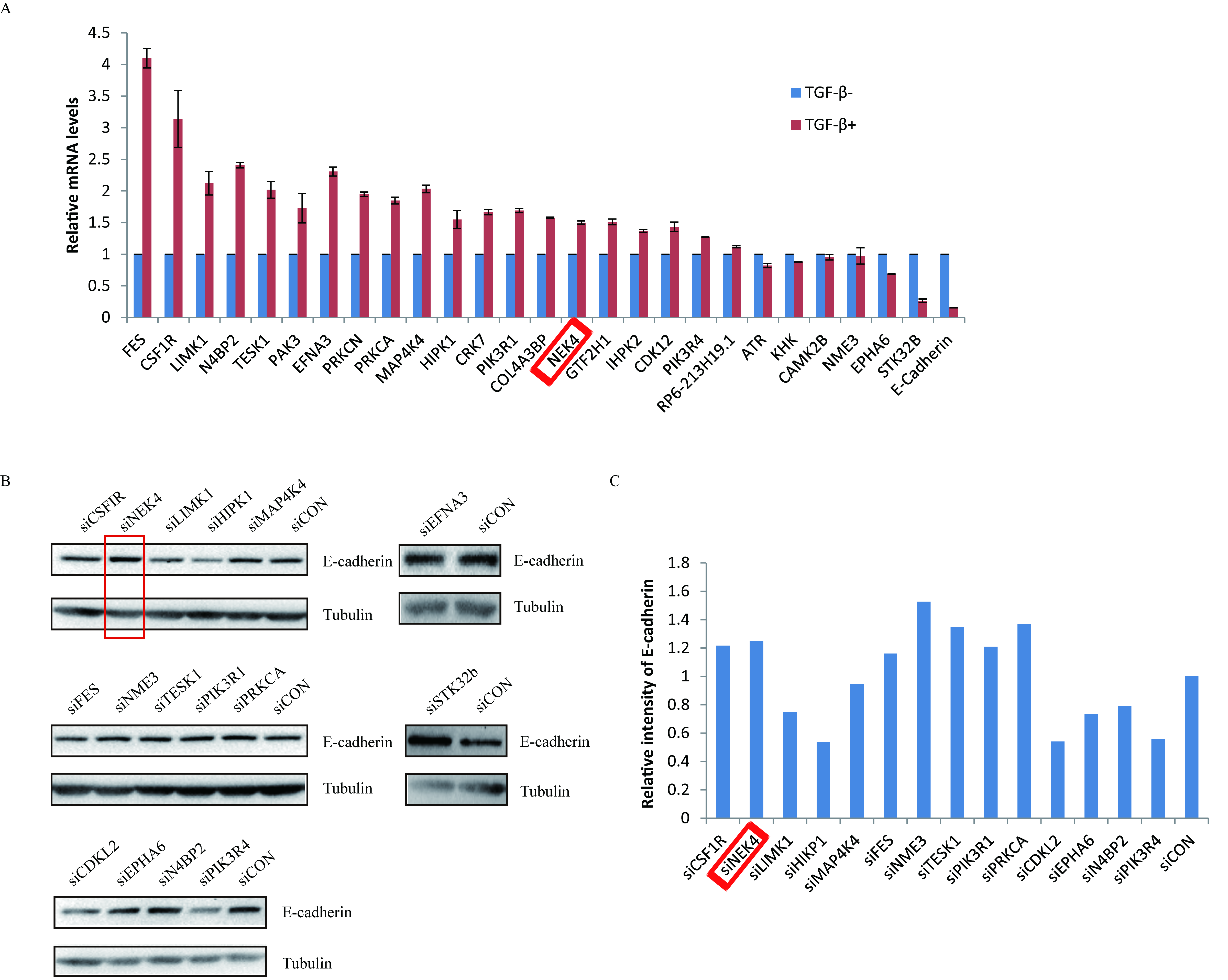

Supplement: Supplementary file 1 [file JCMM-22-5877-s001.tif]

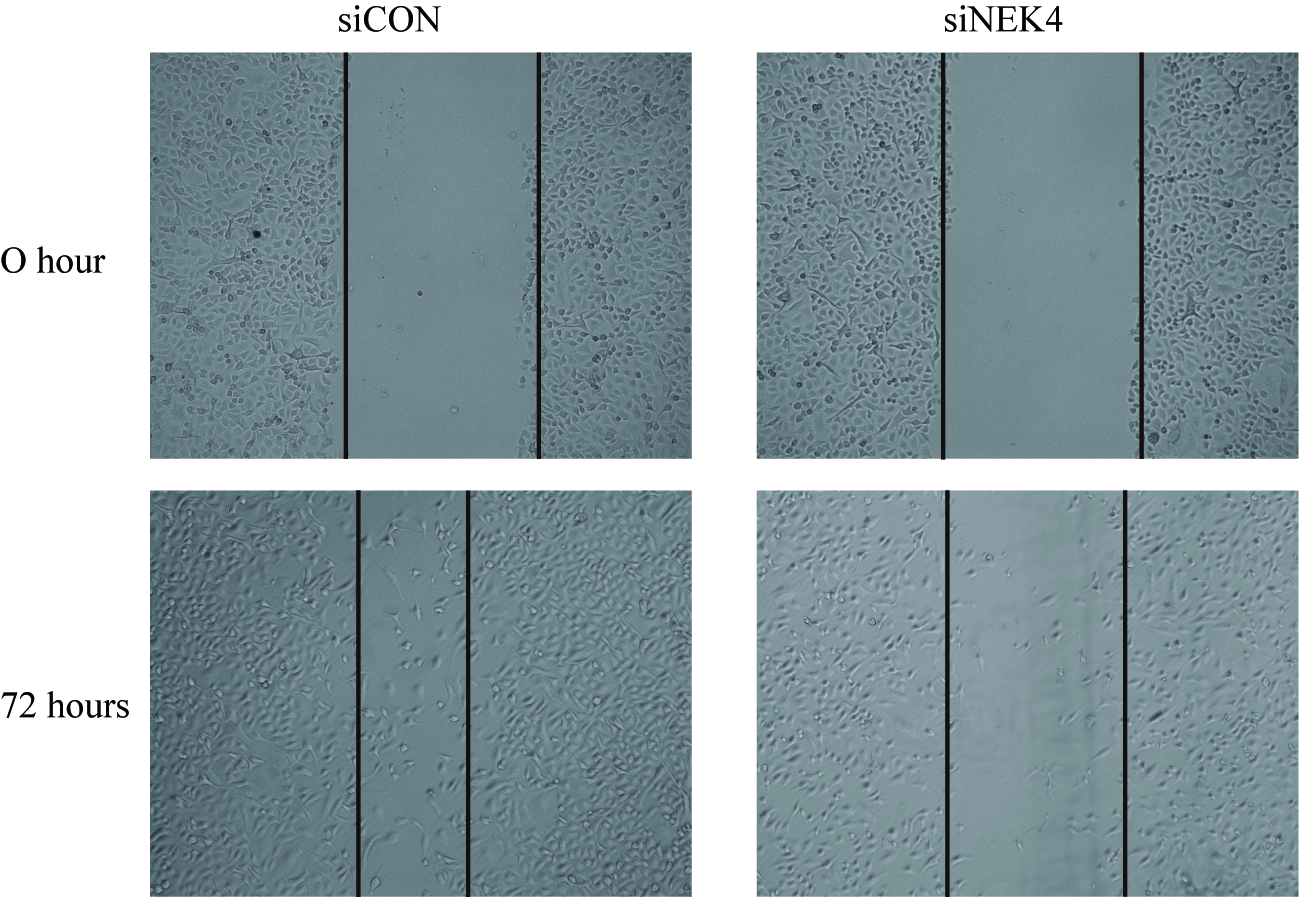

Supplement: Supplementary file 2 [file JCMM-22-5877-s002.tif]

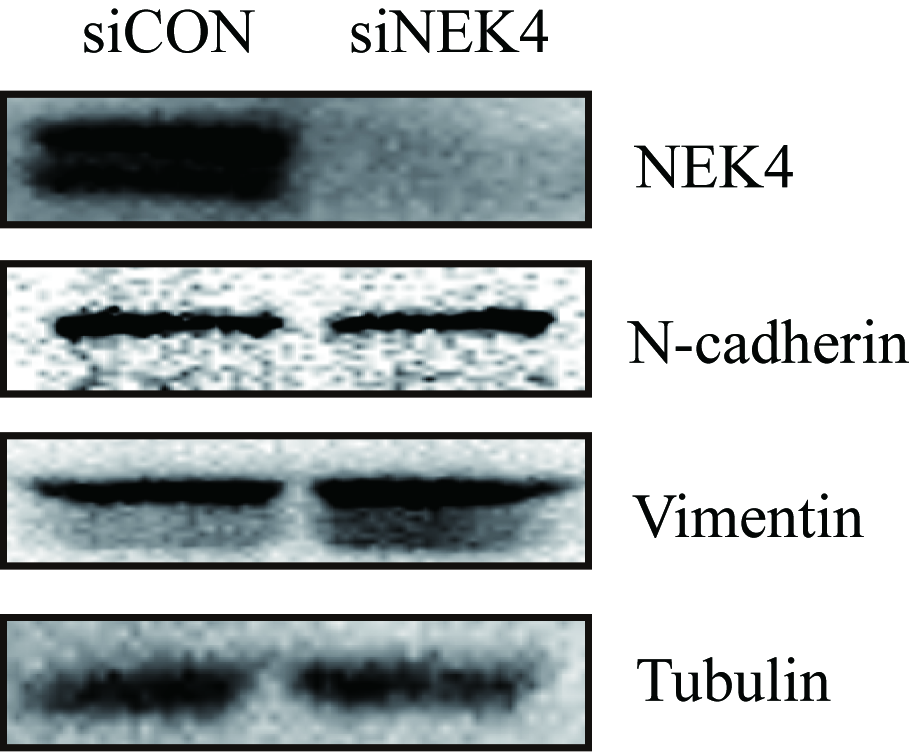

Supplement: Supplementary file 3 [file JCMM-22-5877-s003.tif]

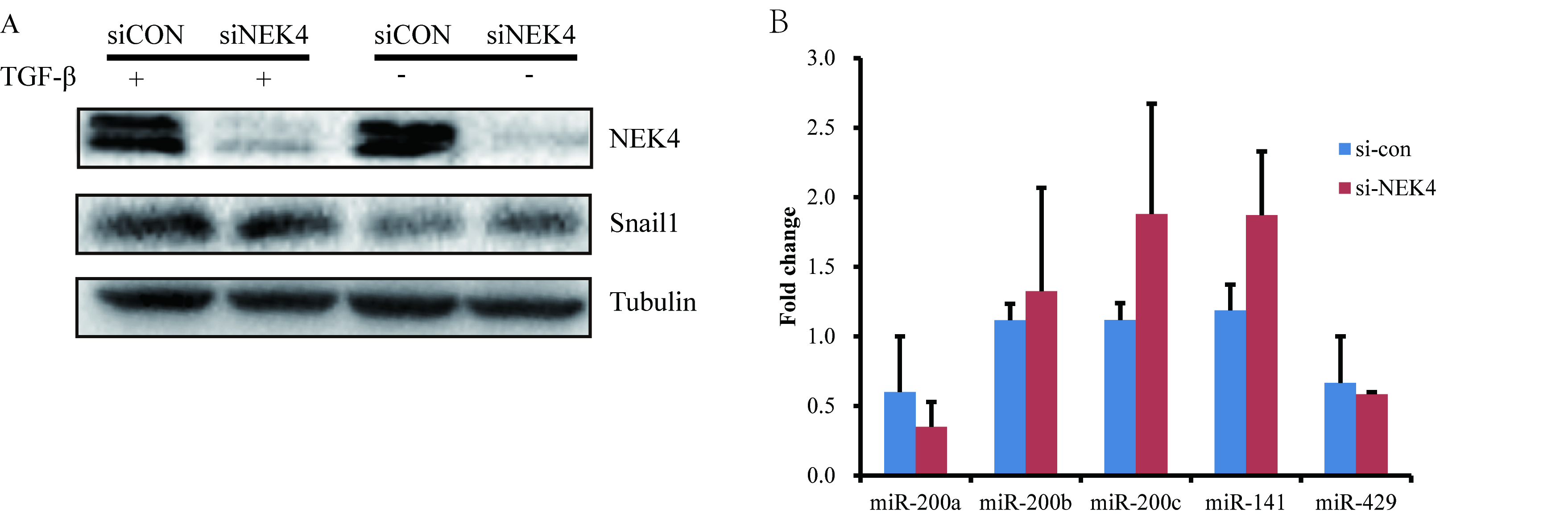

Supplement: Supplementary file 4 [file JCMM-22-5877-s004.tif]
