# Supplementary material for: NEK4 kinase regulates EMT to promote lung cancer metastasis
Source: J Cell Mol Med. 2018 Sep 24;22(12):5877–87. doi: 10.1111/jcmm.13857 (PMC6237562; doi:10.1111/jcmm.13857)
Supplement: Supplementary file 5 [file JCMM-22-5877-s005.docx]

**Supplemental Table 1.**

| **primers sequences** |
| --- |
| **primer sequences for E-cadherin promoter:**  E-cadherin-forward 5’- cggggtaccgccgctcgagcgagagtgcagtgg-3’  E-cadherin-reverse 5’- cgagctcctgcggctccaagggcccatggctg-3’  **primer sequences for shNEK4:**  ShNEK4-forward 5’-GATCCGAACAAACATCATCAAAGTTTCAAGAGAACTTTGATGATGTTTGTTCTTTTTTG-3’  ShNEK4-reverse 5’-AATTCAAAAAAGAACAAACATCATCAAAGTTCTCTTGAAACTTTGATGATGTTTGTTCG-3’  **primer sequences for NEK4:**  NEK4-forward 5’-cccaagcttatgcccctggccgcctactg-3’  NEK4-reverse 5’-ccgctcgagtcaaaaattcatgttttctt-3’  **primer sequences for real-time PCR:**  E-cadherin-forward 5’-ACCACGTACAAGGGTCAGGT-3’  E-cadherin-reverse 5’-GGCATCAGCATCAGTCACTT-3’  CAMK2B-forward 5’-CTCTACGAGGATATTGGCAAGGG-3’  CAMK2B-reverse 5’-GCTTCTGGTGATCTCTGGCTG-3’  NME3-forward 5’-CATCTTCGCTAACCTCTTCCCC-3’  NME3-reverse 5’-CCCGGAGGCCATATACTTGAC-3’  limk1-forward 5’-CAAGGGACTGGTTATGGTGGC-3’  limk1-reverse 5’-CCCCGTCACCGATAAAGGTC-3’  MAP4K4-forward 5’-GACTCCCCTGCAAAAAGTCTG -3’  MAP4K4-reverse 5’-GTCCATAGGTGCCATTTCCAA-3’  PRKCN-forward 5’-CTGCTTCTCCGTGTTCAAGTC -3’  PRKCN-reverse 5’-GAGGCCAATTTGCAGTAGAAATG-3’  NEK4-forward 5’-ACATCATCAAAGTAGGGGACCT-3’  NEK4-reverse 5’-CAGGGCTCATGTAGTAGGGTG-3’  EFNA3-forward 5’-CATGCGGTGTACTGGAACAG-3’  EFNA3-reverse 5’-AGATAGTCGTTCACGTTCACCT-3’  CSF1R –forward 5’-TCCAAAACACGGGGACCTATC-3’  CSF1R -reverse 5’-CGGGCAGGGTCTTTGACATA-3’  HIPK1-forward 5’-TCTCAGTGCCGGAACAAAAAC-3’  HIPK1-reverse 5’-CCCTCCAGGTCTGTAGACATATT-3’  cdk12-forward 5’-CTAACAGCAGAGAGCGTCACC-3’  cdk12-reverse 5’-AAAGGTTTGATAACTGTGCCCA-3’  PIK3R1-forward 5’-TGGACGGCGAAGTAAAGCATT-3’  PIK3R1-reverse 5’-AGTGTGACATTGAGGGAGTCG-3’  PIK3R4-forward 5’-CCTGGTCGTTGTGAAGGTTTT-3’  PIK3R4-reverse 5’-TCTGTGCAGAATTAAGCCTGATT-3’  COL4A3BP-forward 5’-ATGTCGGATAATCAGAGCTGGA-3’  COL4A3BP-reverse 5’-ATCCTGCCACCCATGAATGTA-3’  ATR –forward 5’-GGCCAAAGGCAGTTGTATTGA-3’  ATR -reverse 5’-GTGAGTACCCCAAAAATAGCAGG-3’  STK32B -forward 5’-GGGACTGTGAAACTCTACATCTG-3’  STK32B -reverse 5’-ACCGTCGCTATGTTGAAGTCT-3’  TESK1-forward 5’-GCGCGTGTGGACGATTTTC-3’  TESK1-reverse 5’-GTGCCGAACCTTGTAGACCT-3’  PAK3-forward 5’-CCAGGCTTCGCTCTATCTTCC-3’  PAK3-reverse 5’-TCAAACCCCACATGAATCGTATG-3’  EPHA6-forward 5’-CGCTGTTGGCGGATTCACT-3’  EPHA6-reverse 5’-CGCAAATGCCCATTCTGTAAGT-3’  GTF2H1-forward 5’-GACCTTGTTGTGAGTCAAGTGA-3’  GTF2H1-reverse 5’-CCTGCTTATGATTGGATGTGGAA-3’  N4BP2-forward 5’-CAGTTTTGAACGAGTCCAAGTGT-3’  N4BP2-reverse 5’-CTGCTACAACCTGCGATGGAG-3’  RP6-213H19.1-forward 5’-ATCTTGTGCAAACCCTGAGTTG-3’  RP6-213H19.1-reverse 5’-TTCAATCGCCTGATTCCTGCT-3’  IHPK2-forward 5’-TAACCCTTGGAGCATGAAATGTC-3’  IHPK2-reverse 5’-TCATAGCGGGAAGTCAGGTTT-3’  PRKCA -forward 5’-GTCCACAAGAGGTGCCATGAA-3’  PRKCA -reverse 5’-AAGGTGGGGCTTCCGTAAGT-3’  CDKL2-forward 5’-TCTCCCAGTCTGGCGTTGT-3’  CDKL2-reverse 5’-ACCATCGGGTTGCCACATAAT-3’  FES –forward 5’-AGCTGAAGAGCCAGTACCGA-3’  FES -reverse 5’-GCCTTGTCACGGTCCTTGT-3’  KHK –forward 5’-CTAAGGAGGACTCGGAGATAAGG-3’  KHK -reverse 5’-CATTGAGCCCATGAAGGCAC-3’  **primer sequences for miRNA analysis:**  miR-200a-rt 5’-GTCGTATCCAGTGCAGGGTCCGAGGTATTCGCACTGGATACGACCCAGCA-3’  miR-200a-forward 5’-CCGCGCATCTTACCGGACAG-3’  miR-200a-reverse 5’-ATCCAGTGCAGGGTCCGAGG-3’  miR-200b-rt 5’-GTCGTATCCAGTGCAGGGTCCGAGGTATTCGCACTGGATACGACTCCAAT-3’  miR-200b-forward 5’-CGCGCATCTTACTGGGCAGC-3’  miR-200b-reverse 5’-ATCCAGTGCAGGGTCCGAGG-3’  miR-200c-rt 5’-GTCGTATCCAGTGCAGGGTCCGAGGTATTCGCACTGGATACGACCCAAAC-3’  miR-200c-forward 5’-CGCGCGTCTTACCCAGCAGT-3’  miR-200c-reverse 5’-ATCCAGTGCAGGGTCCGAGG-3’  miR-141-rt 5’-GTCGTATCCAGTGCAGGGTCCGAGGTATTCGCACTGGATACGACTCCAAC-3’  miR-141-forward 5’-CGCGGATCATCTTCCAGTACAGT-3’  miR-141-reverse 5’-ATCCAGTGCAGGGTCCGAGG-3’  miR-419 rt 5’-GTCGTATCCAGTGCAGGGTCCGAGGTATTCGCACTGGATACGACACGGTT-3’  miR-419-forward 5’-CGCGCGGTAATACTGTCTGGTAA-3’  miR-419-reverse 5’-ATCCAGTGCAGGGTCCGAGG-3’ |
