# Supplementary material for: NEK4 kinase regulates EMT to promote lung cancer metastasis
Source: J Cell Mol Med. 2018 Sep 24;22(12):5877–87. doi: 10.1111/jcmm.13857 (PMC6237562; doi:10.1111/jcmm.13857)
Supplement: Supplementary file 6 [file JCMM-22-5877-s006.docx]

**Supplemental Table 2.**

| Plate  number | Plate location | kinases name | fold change of RLU of the first round screening | fold change of RLU of the second round screening |
| --- | --- | --- | --- | --- |
| p1 | a2 | TTK | 0.822000104 |  |
|  | a3 | SYK | 0.996244599 |  |
|  | a4 | EPHA8 | 0.821886931 |  |
|  | a5 | DMPK | 0.590937443 | 0.807497227 |
|  | a6 | COASY | 1.123383339 |  |
|  | a7 | MYO3B | 0.713356123 | 0.509207065 |
|  | a8 | PRKWNK2 | 0.915855684 |  |
|  | a9 | PTK2 | 0.864916051 |  |
|  | a10 | FER | 1.10066648 |  |
|  | a11 | CAMK2B | 0.532619513 | 0.526587779 |
|  | b2 | ACVRL1 | 1.030477621 |  |
|  | b3 | LYN | 0.968348933 |  |
|  | b4 | PAPSS1 | 0.910482724 |  |
|  | b5 | MAPK4 | 0.586180549 | 1.282412699 |
|  | b6 | MASTL | 0.808721017 |  |
|  | b7 | TRIB1 | 0.959864338 |  |
|  | b8 | BRD3 | 0.902046456 |  |
|  | b9 | FLJ34389 | 0.84934747 |  |
|  | b10 | HK1 | 1.015643523 |  |
|  | b11 | THNSL1 | 0.863307237 |  |
|  | c2 | ALS2CR2 | 0.882902745 |  |
|  | c3 | CARKL | 0.580795365 | 0.727056399 |
|  | c4 | ADK | 0.805996155 |  |
|  | c5 | ABL2 | 0.764349725 |  |
|  | c6 | PLK1 | 0.875964822 |  |
|  | c7 | MINK | 1.077184342 |  |
|  | c8 | CDKL3 | 0.916857512 |  |
|  | c9 | MAP3K14 | 0.783909646 |  |
|  | c10 | MAP3K7 | 0.872853445 |  |
|  | c11 | CKB | 0.809612635 |  |
|  | d2 | CDC2 | 0.702231353 | 0.627466213 |
|  | d3 | PIM2 | 0.891062397 |  |
|  | d4 | LRRK2 | 0.748996691 | 1.268905912 |
|  | d5 | HK2 | 0.522391004 | 1.094513403 |
|  | d6 | FN3K | 1.259040108 |  |
|  | d7 | CRIM1 | 0.955244331 |  |
|  | d8 | PIK4CA | 1.136268625 |  |
|  | d9 | SSTK | 0.751404905 |  |
|  | d10 | TAO1 | 0.799089493 |  |
|  | d11 | NME3 | 0.622662596 | 0.349492692 |
|  | e2 | PRKAG3 | 0.520945557 | 1.405721612 |
|  | e3 | GRK5 | 1.069870886 |  |
|  | e4 | LIMK1 | 2.05266154 | 1.989665651 |
|  | e5 | UMPK | 0.856249553 |  |
|  | e6 | C7ORF2 | 0.693421345 | 0.732995156 |
|  | e7 | KSR | 0.745097216 | 0.64656148 |
|  | e8 | MAP4K4 | 0.612011116 | 0.352274577 |
|  | e9 | PRKCN | 0.634806597 | 0.022610041 |
|  | e10 | TTBK1 | 1.645831826 | 0.949267246 |
|  | e11 | ROCK1 | 0.764365722 |  |
|  | f2 | TEK | 1.025703083 |  |
|  | f3 | PANK1 | 2.005275316 | 1.537072621 |
|  | f4 | CSNK1G3 | 0.861362111 |  |
|  | f5 | CKS2 | 1.122384673 |  |
|  | f6 | PHKG2 | 1.256191381 |  |
|  | f7 | MAPK7 | 0.711584849 | 2.054678808 |
|  | f8 | KCNH8 | 1.80207523 | 1.030471059 |
|  | f9 | CAMK2D | 1.031330421 |  |
|  | f10 | MAP4K5 | 1.617694169 | 1.596026026 |
|  | f11 | PKN3 | 1.127092436 |  |
|  | g2 | ATM | 1.341018981 |  |
|  | g3 | PHKA2 | 1.455900067 |  |
|  | g4 | MULK | 0.727348642 | 1.254368729 |
|  | g5 | GRK6 | 1.372515779 |  |
|  | g6 | BMPR1B | 1.288334777 |  |
|  | g7 | STK33 | 0.885532051 |  |
|  | g8 | MIDORI | 0.681038656 | 0.847660702 |
|  | g9 | RNASEL | 1.321733972 |  |
|  | g10 | STK16 | 1.026100729 |  |
|  | g11 | GRK1 | 0.734377911 | 1.080608855 |
|  | h2 | RFP | 0.75950551 |  |
|  | h3 | PIP5KL1 | 1.097778597 |  |
|  | h4 | NEK5 | 0.77003434 |  |
|  | h5 | PLK2 | 0.821856592 |  |
|  | h6 | CSNK1G2 | 1.026285723 |  |
|  | h7 | MARK2 | 1.11063469 |  |
|  | h8 | TNIK | 1.054629905 |  |
|  | h9 | CALM3 | 0.718952207 | 0.994805983 |
|  | h10 | PKM2 | 1.078322337 |  |
|  | h11 | CDKL5 | 1.236847885 |  |
| p2 | a2 | PIK3CA | 1.007178949 |  |
|  | a3 | KIAA1804 | 1.145820876 |  |
|  | a4 | NAGK | 0.960142412 |  |
|  | a5 | IRAK1 | 1.108339866 |  |
|  | a6 | EEF2K | 0.969196994 |  |
|  | a7 | ANKK1 | 0.813338506 |  |
|  | a8 | MAP3K5 | 1.212030493 |  |
|  | a9 | PRKAA1 | 0.931050815 |  |
|  | a10 | RPS6KA4 | 0.708701265 | 2.671711137 |
|  | a11 | CALM1 | 0.809413293 |  |
|  | b2 | FLJ32685 | 0.931874532 |  |
|  | b3 | ITPKC | 1.523689144 |  |
|  | b4 | EPHA5 | 0.88349176 |  |
|  | b5 | MLCK | 1.231038725 |  |
|  | b6 | PSKH1 | 1.108568072 |  |
|  | b7 | AURKC | 0.90431712 |  |
|  | b8 | PRKCE | 0.682656034 | 2.011641326 |
|  | b9 | AK1 | 0.876977869 |  |
|  | b10 | TRPM6 | 1.049230803 |  |
|  | b11 | CLK1 | 1.021397251 |  |
|  | c2 | PAPSS2 | 1.297875604 |  |
|  | c3 | RPS6KB2 | 1.16055023 |  |
|  | c4 | PFTK1 | 1.215640447 |  |
|  | c5 | NME5 | 1.122918062 |  |
|  | c6 | SRC | 1.257849828 |  |
|  | c7 | NEK4 | 1.954152749 | 2.361757721 |
|  | c8 | CSNK1G1 | 1.031752772 |  |
|  | c9 | RIOK1 | 1.065861413 |  |
|  | c10 | AKT1 | 1.012268898 |  |
|  | c11 | SNRK | 1.091579002 |  |
|  | d2 | KIAA1811 | 1.132014761 |  |
|  | d3 | PDK2 | 1.136367619 |  |
|  | d4 | MYLK2 | 1.051572597 |  |
|  | d5 | NEK11 | 1.095091169 |  |
|  | d6 | KIAA1361 | 0.959342757 |  |
|  | d7 | SNF1LK | 1.017156332 |  |
|  | d8 | PTK9 | 1.155779188 |  |
|  | d9 | LATS1 | 1.160536848 |  |
|  | d10 | PFKP | 0.933526926 |  |
|  | d11 | PRKG1 | 1.118969919 |  |
|  | e2 | RIOK2 | 1.196241085 |  |
|  | e3 | FRAP1 | 1.764255182 | 1.485947028 |
|  | e4 | PDGFRL | 1.254879977 |  |
|  | e5 | AK7 | 1.780798318 | 0.51108013 |
|  | e6 | EPHA10 | 0.762448344 |  |
|  | e7 | STK17B | 0.986594381 |  |
|  | e8 | NPR2 | 1.008636941 |  |
|  | e9 | GOLGA5 | 1.047833983 |  |
|  | e10 | STK24 | 1.066022675 |  |
|  | e11 | DGKB | 1.100209461 |  |
|  | f2 | STK25 | 0.960477112 |  |
|  | f3 | CDC7 | 1.19469528 |  |
|  | f4 | CAMK2G | 1.113433831 |  |
|  | f5 | URKL1 | 1.377580668 |  |
|  | f6 | MAP3K9 | 1.069560746 |  |
|  | f7 | GUCY2D | 1.299399011 |  |
|  | f8 | CAMKK2 | 1.089208907 |  |
|  | f9 | KIAA1639 | 1.32698827 |  |
|  | f10 | CAMKIINALPHA | 0.914456241 |  |
|  | f11 | KIT | 1.022830118 |  |
|  | g2 | PRKCL1 | 1.090638194 |  |
|  | g3 | RIPK2 | 0.956622822 |  |
|  | g4 | CKMT1B | 0.987063432 |  |
|  | g5 | MKNK2 | 0.995354472 |  |
|  | g6 | TNNI3K | 0.939876909 |  |
|  | g7 | ALS2CR7 | 1.111578927 |  |
|  | g8 | HCK | 0.859427521 |  |
|  | g9 | CKS1B | 0.904437893 |  |
|  | g10 | BMP2K | 0.748100093 | 0.861743949 |
|  | g11 | PHKB | 1.060771453 |  |
|  | h2 | TRIO | 0.930866835 |  |
|  | h3 | ETNK1 | 0.857988335 |  |
|  | h4 | ERK8 | 0.87875571 |  |
|  | h5 | ADP-GK | 1.442923135 |  |
|  | h6 | PIK3CG | 0.947812922 |  |
|  | h7 | FGFR3 | 1.154109162 |  |
|  | h8 | IRAK4 | 1.009514973 |  |
|  | h9 | PCTK3 | 0.975918023 |  |
|  | h10 | SGK2 | 0.838924405 |  |
|  | h11 | DYRK4 | 0.793700186 |  |
| p3 | a2 | MAP3K2 | 0.970046182 |  |
|  | a3 | LATS2 | 1.147738305 |  |
|  | a4 | P101-PI3K | 1.229341159 |  |
|  | a5 | DGKG | 1.34504665 |  |
|  | a6 | MAST3 | 1.244281679 |  |
|  | a7 | EFNA3 | 2.035399948 | 4.871943837 |
|  | a8 | BCKDK | 0.634587217 | 0.813649483 |
|  | a9 | ITPKA | 0.834666373 |  |
|  | a10 | NEK6 | 1.543106025 |  |
|  | a11 | BLK | 1.022321711 |  |
|  | b2 | DGKK | 1.079146548 |  |
|  | b3 | MPP2 | 0.989321301 |  |
|  | b4 | BUB1 | 0.739671404 | 1.837456238 |
|  | b5 | UHMK1 | 0.981244099 |  |
|  | b6 | PRKCM | 1.149406068 |  |
|  | b7 | PIK4CB | 1.302342247 |  |
|  | b8 | DLG4 | 1.259954409 |  |
|  | b9 | BTK | 0.901279933 |  |
|  | b10 | MAP3K15 | 0.980628199 |  |
|  | b11 | GSK3A | 1.138520171 |  |
|  | c2 | CSNK1E | 1.273075265 |  |
|  | c3 | RELA | 0.960868368 |  |
|  | c4 | CCRK | 0.848603 |  |
|  | c5 | MAPK9 | 1.105416931 |  |
|  | c6 | TNK2 | 0.876647464 |  |
|  | c7 | PDIK1L | 1.039249344 |  |
|  | c8 | AXL | 1.097636926 |  |
|  | c9 | ANKRD3 | 1.242770257 |  |
|  | c10 | RIPK1 | 0.92504181 |  |
|  | c11 | CDC42BPB | 0.738931372 | 1.660845848 |
|  | d2 | JAK3 | 0.913641495 |  |
|  | d3 | STK22D | 0.727888996 | 0.581102023 |
|  | d4 | FGFR1 | 1.040868513 |  |
|  | d5 | PNKP | 1.221382882 |  |
|  | d6 | ERBB2 | 0.80854704 |  |
|  | d7 | PRKAG1 | 0.911812509 |  |
|  | d8 | FLJ10761 | 0.815380748 |  |
|  | d9 | IGF1R | 1.033249075 |  |
|  | d10 | NEK9 | 0.821975564 |  |
|  | d11 | FLJ23356 | 0.925577367 |  |
|  | e2 | FLT3 | 1.10432783 |  |
|  | e3 | PIP5K2A | 1.028417359 |  |
|  | e4 | DAPK1 | 1.059006001 |  |
|  | e5 | FASTK | 1.144340896 |  |
|  | e6 | PRKG2 | 1.234336541 |  |
|  | e7 | MPP1 | 1.087203952 |  |
|  | e8 | HSPB8 | 0.921438188 |  |
|  | e9 | CSF1R | 0.646831526 | 0.576251422 |
|  | e10 | DYRK2 | 1.085424625 |  |
|  | e11 | PRKCL2 | 0.965227427 |  |
|  | f2 | RPS6KA3 | 0.956138429 |  |
|  | f3 | EFNA4 | 1.353354069 |  |
|  | f4 | ADCK1 | 1.048104815 |  |
|  | f5 | MAP3K8 | 1.111194386 |  |
|  | f6 | CDK8 | 1.343166763 |  |
|  | f7 | MKNK1 | 0.916549864 |  |
|  | f8 | PIK3C2G | 1.193166651 |  |
|  | f9 | CDK4 | 1.057941082 |  |
|  | f10 | PIM1 | 0.986055293 |  |
|  | f11 | DGKQ | 1.221935778 |  |
|  | g2 | MAP3K12 | 1.292040924 |  |
|  | g3 | STK22C | 1.565583069 |  |
|  | g4 | PI4KII | 1.065886152 |  |
|  | g5 | TAF1L | 1.189680362 |  |
|  | g6 | CAMK1G | 1.183449157 |  |
|  | g7 | HSMDPKIN | 0.991749309 |  |
|  | g8 | STK10 | 0.949704874 |  |
|  | g9 | AIP1 | 1.076655184 |  |
|  | g10 | ABL1 | 1.294268138 |  |
|  | g11 | PAK6 | 1.028864216 |  |
|  | h2 | FN3KRP | 0.74663673 | 0.451833501 |
|  | h3 | AKT2 | 1.275078696 |  |
|  | h4 | KIAA0999 | 0.933911582 |  |
|  | h5 | PLK4 | 0.983696579 |  |
|  | h6 | HIPK3 | 1.123842904 |  |
|  | h7 | TYRO3 | 0.922541384 |  |
|  | h8 | MAPK3 | 1.054884861 |  |
|  | h9 | YES1 | 0.950511271 |  |
|  | h10 | NYD-SP25 | 1.235192708 |  |
|  | h11 | CDADC1 | 1.4380559 |  |
| p4 | a2 | STK31 | 0.998763242 |  |
|  | a3 | AKT3 | 1.370263365 |  |
|  | a4 | NLK | 0.971889531 |  |
|  | a5 | PFKFB3 | 1.099957637 |  |
|  | a6 | TESK2 | 0.643103229 | 1.582508639 |
|  | a7 | EPHA7 | 0.969002028 |  |
|  | a8 | STK35 | 1.263352589 |  |
|  | a9 | STK11 | 0.946189758 |  |
|  | a10 | JAK2 | 0.801238809 |  |
|  | a11 | BRD2 | 0.901589798 |  |
|  | b2 | CDKN2C | 0.867570048 |  |
|  | b3 | MAP2K3 | 0.827963676 |  |
|  | b4 | LCK | 0.976473995 |  |
|  | b5 | CIB2 | 0.852314332 |  |
|  | b6 | AAK1 | 0.712326845 | 1.09318692 |
|  | b7 | PIK3R2 | 0.973295546 |  |
|  | b8 | IRAK2 | 0.929744164 |  |
|  | b9 | ACVR1C | 1.065341209 |  |
|  | b10 | ACVR1B | 1.541411326 |  |
|  | b11 | CPNE3 | 0.909893279 |  |
|  | c2 | CAMK1 | 1.210024049 |  |
|  | c3 | MARK3 | 0.790612751 |  |
|  | c4 | MGC42105 | 0.755706512 |  |
|  | c5 | CHEK1 | 0.666320789 | 1.960127942 |
|  | c6 | SCAP1 | 0.972215176 |  |
|  | c7 | CDC2L2 | 0.840987306 |  |
|  | c8 | STK3 | 1.050390425 |  |
|  | c9 | FLT4 | 0.693675109 | 0.808595219 |
|  | c10 | CDC2L1 | 0.952263765 |  |
|  | c11 | MAP2K7 | 1.031824653 |  |
|  | d2 | EPHB6 | 1.385911142 |  |
|  | d3 | MARK1 | 0.958680019 |  |
|  | d4 | PRKAR2A | 0.734369666 | 1.884458563 |
|  | d5 | PINK1 | 1.027915543 |  |
|  | d6 | MUSK | 1.099087571 |  |
|  | d7 | ULK2 | 0.808576328 |  |
|  | d8 | MGC8407 | 1.003560553 |  |
|  | d9 | AURKA | 1.214318056 |  |
|  | d10 | PAK4 | 0.564675064 | 1.065774094 |
|  | d11 | MAPK1 | 0.832816265 |  |
|  | e2 | MAST4 | 0.871185145 |  |
|  | e3 | CKMT2 | 0.818878822 |  |
|  | e4 | PIK3R3 | 0.895008555 |  |
|  | e5 | ACVR2 | 0.789102413 |  |
|  | e6 | PRKWNK3 | 0.978230569 |  |
|  | e7 | PRKCZ | 0.947315498 |  |
|  | e8 | ITK | 0.67105537 | 0.905316207 |
|  | e9 | CAMKK1 | 1.040899236 |  |
|  | e10 | STK36 | 0.793532352 |  |
|  | e11 | MAPK8 | 0.701022283 | 0.917830758 |
|  | f2 | TGFBR1 | 0.837668794 |  |
|  | f3 | CDK5R2 | 1.203978217 |  |
|  | f4 | LOC91461 | 1.288960667 |  |
|  | f5 | DKFZP761P0423 | 0.752859337 |  |
|  | f6 | DUSTYPK | 1.32222397 |  |
|  | f7 | FLJ23356 | 0.742600932 | 0.993684134 |
|  | f8 | PDK3 | 0.836093448 |  |
|  | f9 | DGKA | 1.040806723 |  |
|  | f10 | C9ORF12 | 1.070128568 |  |
|  | f11 | PTK6 | 1.28056396 |  |
|  | g2 | PAK1 | 0.735747423 | 1.161722399 |
|  | g3 | RYK | 0.848065198 |  |
|  | g4 | PDXK | 1.161966634 |  |
|  | g5 | NEK8 | 0.960051302 |  |
|  | g6 | GK | 1.030967674 |  |
|  | g7 | KSR2 | 0.790662589 |  |
|  | g8 | PRKAR1A | 0.845676513 |  |
|  | g9 | NUP62 | 1.861213451 | 1.484210397 |
|  | g10 | ROR2 | 1.304826334 |  |
|  | g11 | PIP5K2C | 1.13416792 |  |
|  | h2 | ACVR1 | 0.920736873 |  |
|  | h3 | PRKACB | 0.829705249 |  |
|  | h4 | STK39 | 0.87945716 |  |
|  | h5 | MATK | 1.220309536 |  |
|  | h6 | RPS6KB1 | 1.112501678 |  |
|  | h7 | RFK | 1.078530984 |  |
|  | h8 | TTBK2 | 0.882765847 |  |
|  | h9 | CDC42BPA | 0.846378092 |  |
|  | h10 | GSG2 | 0.862231945 |  |
|  | h11 | HIPK1 | 1.788518331 | 1.635543279 |
| p5 | a2 | MAP3K11 | 0.806463813 |  |
|  | a3 | AURKB | 1.163524109 |  |
|  | a4 | LIMK2 | 0.565217587 | 2.210738799 |
|  | a5 | GRK4 | 0.850097751 |  |
|  | a6 | LOC340156 | 1.243528968 |  |
|  | a7 | PRKX | 0.536801272 | 2.013396975 |
|  | a8 | GNE | 1.252385351 |  |
|  | a9 | PRKAR1B | 1.32607498 |  |
|  | a10 | MVK | 1.135952967 |  |
|  | a11 | PKIB | 0.852833001 |  |
|  | b2 | CDK5 | 1.016304366 |  |
|  | b3 | ADCK4 | 1.488679244 |  |
|  | b4 | HRI | 1.222784392 |  |
|  | b5 | MAPKAPK3 | 0.786983837 |  |
|  | b6 | ACVR2B | 0.825076564 |  |
|  | b7 | EGFR | 0.915929906 |  |
|  | b8 | ROS1 | 0.660552203 | 1.955528341 |
|  | b9 | HK3 | 1.304915342 |  |
|  | b10 | DDR2 | 1.170955024 |  |
|  | b11 | DAPK2 | 0.911196993 |  |
|  | c2 | ITPK1 | 1.205429062 |  |
|  | c3 | ILK-2 | 1.058329876 |  |
|  | c4 | EPHA2 | 1.181711829 |  |
|  | c5 | PHKG1 | 0.875330944 |  |
|  | c6 | FUK | 1.216593483 |  |
|  | c7 | AK3 | 1.252727099 |  |
|  | c8 | CSNK2A2 | 0.951005283 |  |
|  | c9 | VRK2 | 1.234147266 |  |
|  | c10 | KALRN | 1.041433538 |  |
|  | c11 | ARAF1 | 1.202666746 |  |
|  | d2 | BCR | 1.469786506 |  |
|  | d3 | DCK | 1.224574176 |  |
|  | d4 | MGC16169 | 1.076792994 |  |
|  | d5 | STK22D | 0.689231783 | 1.478712712 |
|  | d6 | SRP72 | 0.885291204 |  |
|  | d7 | DGKI | 1.143602388 |  |
|  | d8 | CSK | 1.119470879 |  |
|  | d9 | IKBKAP | 0.824658879 |  |
|  | d10 | ADRBK2 | 0.862575599 |  |
|  | d11 | ICK | 1.388894951 |  |
|  | e2 | ADCK2 | 1.239035812 |  |
|  | e3 | PI4K2B | 1.131756931 |  |
|  | e4 | TRIB3 | 1.504844926 |  |
|  | e5 | FLJ23074 | 1.40737569 |  |
|  | e6 | HIPK2 | 0.898266036 |  |
|  | e7 | PRPS1 | 1.342527806 |  |
|  | e8 | CSNK1D | 0.800707405 |  |
|  | e9 | DYRK3 | 1.496091269 |  |
|  | e10 | TYK2 | 0.98279354 |  |
|  | e11 | MYO3A | 1.852580706 | 1.344644649 |
|  | f2 | SPHK1 | 1.12276145 |  |
|  | f3 | ASK | 0.882282025 |  |
|  | f4 | PDGFRB | 1.033534736 |  |
|  | f5 | FGR | 1.30972489 |  |
|  | f6 | ROR1 | 1.187456023 |  |
|  | f7 | CRK7 | 2.44695532 | 2.302711147 |
|  | f8 | CDKN1B | 0.866657542 |  |
|  | f9 | PACSIN1 | 1.179393209 |  |
|  | f10 | DAPK3 | 1.009441479 |  |
|  | f11 | PIK3R1 | 1.536547185 | 2.538819582 |
|  | g2 | PRKY | 0.99161415 |  |
|  | g3 | MAP3K1 | 0.853046233 |  |
|  | g4 | ZAP70 | 1.618468159 | 0.703540616 |
|  | g5 | STK23 | 1.181443043 |  |
|  | g6 | PTK7 | 1.352707645 |  |
|  | g7 | RPS6KL1 | 1.065026095 |  |
|  | g8 | RPS6KA6 | 1.369847027 |  |
|  | g9 | SRPK1 | 1.404555205 |  |
|  | g10 | PRKACA | 0.852851958 |  |
|  | g11 | PTK2B | 1.693455252 | 1.293779805 |
|  | h2 | MERTK | 1.334740068 |  |
|  | h3 | CERK | 0.972983617 |  |
|  | h4 | NUCKS | 1.250162408 |  |
|  | h5 | CIT | 0.957215077 |  |
|  | h6 | CDKL4 | 1.012648391 |  |
|  | h7 | KIAA2002 | 1.021285231 |  |
|  | h8 | OSR1 | 1.123798405 |  |
|  | h9 | RAGE | 0.867029762 |  |
|  | h10 | BMPR2 | 1.335490168 |  |
|  | h11 | NRBP2 | 0.738341759 | 1.606698442 |
| p6 | a2 | MAP2K4 | 1.142003366 |  |
|  | a3 | FLJ13052 | 0.959916682 |  |
|  | a4 | TLK2 | 1.390368493 |  |
|  | a5 | TJP2 | 1.245657418 |  |
|  | a6 | ADCK5 | 1.039877955 |  |
|  | a7 | MAPK11 | 0.914435381 |  |
|  | a8 | PGK2 | 1.394949654 |  |
|  | a9 | BUB1B | 0.734738809 | 1.095374717 |
|  | a10 | MAP2K5 | 0.934911636 |  |
|  | a11 | LAK | 1.290310565 |  |
|  | b2 | CDK7 | 0.857442541 |  |
|  | b3 | LRRK1 | 1.042603226 |  |
|  | b4 | FLJ25006 | 0.800063738 |  |
|  | b5 | MAPK14 | 0.687611865 | 2.137798879 |
|  | b6 | TBK1 | 0.889883424 |  |
|  | b7 | ERN2 | 0.800791006 |  |
|  | b8 | PRKCI | 0.805597408 |  |
|  | b9 | IKBKE | 1.068614037 |  |
|  | b10 | CSNK1A1L | 0.868321038 |  |
|  | b11 | PIP5K1A | 0.924469029 |  |
|  | c2 | STK17A | 1.247902935 |  |
|  | c3 | TEX14 | 1.049471575 |  |
|  | c4 | MAPKAPK2 | 0.885639867 |  |
|  | c5 | LTK | 1.472638872 |  |
|  | c6 | CASK | 1.056425686 |  |
|  | c7 | DYRK1B | 1.056580132 |  |
|  | c8 | SBK1 | 0.718127027 | 2.2527271 |
|  | c9 | PCTK1 | 1.013275521 |  |
|  | c10 | MAP3K4 | 0.915252312 |  |
|  | c11 | STK38L | 1.153025686 |  |
|  | d2 | MGC4796 | 1.426559556 |  |
|  | d3 | PKIA | 1.017150398 |  |
|  | d4 | RBKS | 1.174282122 |  |
|  | d5 | PHKA1 | 0.870015847 |  |
|  | d6 | ERN1 | 0.682773177 | 0.954173239 |
|  | d7 | STYK1 | 0.557836096 | 3.014353836 |
|  | d8 | ARK5 | 0.757238378 |  |
|  | d9 | PIP5K1B | 0.94094078 |  |
|  | d10 | DGUOK | 0.951758422 |  |
|  | d11 | PANK3 | 0.922103319 |  |
|  | e2 | SRPK2 | 1.141180574 |  |
|  | e3 | CLK2 | 0.707971265 | 1.542112506 |
|  | e4 | TGFBR2 | 0.951025185 |  |
|  | e5 | STK22B | 1.225783619 |  |
|  | e6 | GAK | 1.084556511 |  |
|  | e7 | RPS6KA2 | 2.146697155 | 1.590560992 |
|  | e8 | MAPK6 | 0.741588519 | 2.189200106 |
|  | e9 | GRK7 | 0.874521497 |  |
|  | e10 | ADRBK1 | 0.645758038 | 2.914194354 |
|  | e11 | CDK10 | 1.272969115 |  |
|  | f2 | CDKN1A | 0.97704439 |  |
|  | f3 | PANK2 | 1.050265206 |  |
|  | f4 | EPHA3 | 0.646852592 | 0.817001339 |
|  | f5 | PYCS | 0.773600714 |  |
|  | f6 | IHPK1 | 1.197136061 |  |
|  | f7 | BRD4 | 0.995225755 |  |
|  | f8 | TEC | 1.39589088 |  |
|  | f9 | FRDA | 0.92319118 |  |
|  | f10 | PRPS2 | 1.057478083 |  |
|  | f11 | AK5 | 1.025465323 |  |
|  | g2 | MAST2 | 0.84344632 |  |
|  | g3 | CDK5R1 | 2.185265153 | 0.668630076 |
|  | g4 | WEE1 | 1.16929494 |  |
|  | g5 | CDK6 | 0.831194706 |  |
|  | g6 | CDKN2D | 1.324319831 |  |
|  | g7 | PRKCQ | 0.72260476 | 0.775368139 |
|  | g8 | PRKAG2 | 0.802547589 |  |
|  | g9 | MAP3K3 | 1.043969762 |  |
|  | g10 | FGFRL1 | 1.096197918 |  |
|  | g11 | NME1 | 0.81675598 |  |
|  | h2 | TNK1 | 1.137232702 |  |
|  | h3 | STK29 | 0.859126501 |  |
|  | h4 | NTRK3 | 1.35193601 |  |
|  | h5 | LMTK2 | 0.96425655 |  |
|  | h6 | DTYMK | 1.173451684 |  |
|  | h7 | PIK3R4 | 0.407616622 | 0.56195151 |
|  | h8 | GCK | 0.870644213 |  |
|  | h9 | EPHB2 | 0.876698338 |  |
|  | h10 | SGKL | 1.039139009 |  |
|  | h11 | DLG3 | 1.013071481 |  |
| p7 | a2 | RET | 1.140241368 |  |
|  | a3 | MAP2K6 | 0.643553192 | 1.888888889 |
|  | a4 | RPS6KC1 | 1.016322424 |  |
|  | a5 | NME7 | 0.949378707 |  |
|  | a6 | MAP3K10 | 0.760148627 |  |
|  | a7 | UCK1 | 1.403913852 |  |
|  | a8 | FGFR2 | 1.063068595 |  |
|  | a9 | ERBB3 | 0.842037999 |  |
|  | a10 | NEK3 | 0.588783304 | 0.855421687 |
|  | a11 | COL4A3BP | 0.528708327 | 0.563587684 |
|  | b2 | CDC2L5 | 1.237443053 |  |
|  | b3 | ALK | 1.095577319 |  |
|  | b4 | SPEG | 0.589657978 | 0.812392427 |
|  | b5 | TP53RK | 0.609407793 | 0.953145917 |
|  | b6 | EFNA5 | 1.171808602 |  |
|  | b7 | MAPK13 | 0.922142964 |  |
|  | b8 | TRIB2 | 1.10887925 |  |
|  | b9 | DGKH | 0.899270349 |  |
|  | b10 | FRK | 0.94521789 |  |
|  | b11 | PIK3CB | 0.760923932 |  |
|  | c2 | KCNH2 | 0.894218184 |  |
|  | c3 | CDKL1 | 0.723251901 | 0.792503347 |
|  | c4 | NTRK2 | 0.663612467 | 0.63415567 |
|  | c5 | AATK | 0.649496666 | 0.993395805 |
|  | c6 | PRKCG | 0.618005993 | 1.331229681 |
|  | c7 | STK38 | 0.639041806 | 0.665774208 |
|  | c8 | PMVK | 0.704860485 | 0.686746988 |
|  | c9 | EPHA1 | 0.822282802 |  |
|  | c10 | RAF1 | 1.006904432 |  |
|  | c11 | PRKCSH | 0.818781519 |  |
|  | d2 | PRKCD | 0.857808179 |  |
|  | d3 | ATR | 0.487840593 | 0.609705489 |
|  | d4 | CAMK2A | 0.805747573 |  |
|  | d5 | KDR | 1.055606891 |  |
|  | d6 | TAF1 | 0.616973491 | 2.200803213 |
|  | d7 | CDK11 | 1.18790708 |  |
|  | d8 | SNARK | 0.669998689 | 1.487728693 |
|  | d9 | SCYL1 | 0.841646916 |  |
|  | d10 | PRKAB2 | 0.958419963 |  |
|  | d11 | MGC4796 | 1.075486251 |  |
|  | e2 | STK32B | 0.487465922 | 0.497227003 |
|  | e3 | MAP3K7IP1 | 0.878218928 |  |
|  | e4 | PRKR | 0.868654616 |  |
|  | e5 | RIPK3 | 0.71157267 | 1.002409639 |
|  | e6 | ULK4 | 0.73286918 | 0.378045515 |
|  | e7 | SPHK2 | 0.616082972 | 0.364304484 |
|  | e8 | TESK1 | 0.427012287 | 0.608537855 |
|  | e9 | CSNK2B | 0.647579959 | 0.613501626 |
|  | e10 | ERBB4 | 0.769580156 |  |
|  | e11 | PFKFB4 | 0.564508664 | 1.617567349 |
|  | f2 | PKMYT1 | 0.709791671 | 2.50900867 |
|  | f3 | PACE-1 | 0.946740787 |  |
|  | f4 | IKBKB | 0.5576856 | 1.037912773 |
|  | f5 | MAP3K13 | 0.587790504 | 4.813813163 |
|  | f6 | CAMK1D | 0.775971583 |  |
|  | f7 | IRAK3 | 0.822028898 |  |
|  | f8 | MOS | 0.780156931 |  |
|  | f9 | PIP5K2B | 0.526213333 | 1.108508359 |
|  | f10 | STK4 | 0.762684742 |  |
|  | f11 | BAIAP1 | 1.061921274 |  |
|  | g2 | SLK | 0.882021733 |  |
|  | g3 | CDKN1C | 0.913238865 |  |
|  | g4 | PIP5K1C | 0.798831277 |  |
|  | g5 | PFKL | 0.874489658 |  |
|  | g6 | PCK1 | 0.569353484 | 2.547747403 |
|  | g7 | CDK9 | 0.559458579 | 2.837837454 |
|  | g8 | FGFR4 | 0.74427316 | 1.657094371 |
|  | g9 | CRKL | 0.309868495 | 0.86186161 |
|  | g10 | RPS6KA5 | 0.557779722 | 1.867526365 |
|  | g11 | STK32C | 0.79364827 |  |
|  | h2 | PRKD2 | 0.82837197 |  |
|  | h3 | CAMKK1 | 1.026321356 |  |
|  | h4 | LYK5 | 1.060184052 |  |
|  | h5 | COMMD3 | 0.498867015 | 0.745495395 |
|  | h6 | TK2 | 0.805890605 |  |
|  | h7 | CSNK2A1 | 0.869689196 |  |
|  | h8 | SRMS | 0.820975624 |  |
|  | h9 | MAP4K2 | 0.673404527 | 2.043242967 |
|  | h10 | KIAA1811 | 0.9731454 |  |
|  | h11 | PDK1 | 0.717017123 | 2.007507236 |
| p8 | a2 | MELK | 1.100857338 |  |
|  | a3 | PRKCH | 0.458009204 | 1.312189352 |
|  | a4 | PAK3 | 0.599464238 | 0.554525223 |
|  | a5 | PFKFB1 | 1.334611021 |  |
|  | a6 | INSRR | 1.395914089 |  |
|  | a7 | DLG2 | 1.040863683 |  |
|  | a8 | PIP5K3 | 1.377915737 |  |
|  | a9 | BMPR1A | 0.968127206 |  |
|  | a10 | PRPS1L1 | 1.045652436 |  |
|  | a11 | IGF2R | 1.206975008 |  |
|  | b2 | TGFBR3 | 0.870920481 |  |
|  | b3 | UMP-CMPK | 0.647255274 | 0.675773019 |
|  | b4 | CHKA | 0.767315769 |  |
|  | b5 | WNK4 | 1.612713739 | 1.489474603 |
|  | b6 | RPS6KA1 | 1.805729543 | 2.157344214 |
|  | b7 | IHPK3 | 1.14305779 |  |
|  | b8 | BMX | 1.024231348 |  |
|  | b9 | CLK4 | 1.110354792 |  |
|  | b10 | PAK2 | 1.076660523 |  |
|  | b11 | LOC390226 | 1.278496314 |  |
|  | c2 | CSNK1A1 | 1.440765918 |  |
|  | c3 | NME2 | 1.144267604 |  |
|  | c4 | MAK | 0.75707934 |  |
|  | c5 | PIK3CD | 1.17428423 |  |
|  | c6 | MAP3K6 | 0.762329104 |  |
|  | c7 | BRDT | 0.739380816 | 1.475652038 |
|  | c8 | EIF2AK4 | 0.715282165 | 0.838649852 |
|  | c9 | NME6 | 0.680736019 | 0.713265559 |
|  | c10 | DYRK1A | 0.700719509 | 0.86927939 |
|  | c11 | CAMK4 | 1.016280218 |  |
|  | d2 | JIK | 0.732640752 | 0.551764552 |
|  | d3 | PKLR | 1.391989708 |  |
|  | d4 | PAK7 | 0.839954658 |  |
|  | d5 | VRK1 | 0.766150989 |  |
|  | d6 | EPHA6 | 0.372390707 | 0.534189137 |
|  | d7 | GUCY2C | 0.777878857 |  |
|  | d8 | PNCK | 1.075555376 |  |
|  | d9 | VRK3 | 0.980430697 |  |
|  | d10 | PDGFRA | 0.879989554 |  |
|  | d11 | DDR1 | 1.398113698 |  |
|  | e2 | PTK9L | 0.758131523 |  |
|  | e3 | PIK3C2A | 0.635963433 | 0.545513388 |
|  | e4 | STK32A | 0.902168981 |  |
|  | e5 | GTF2H1 | 0.484296461 | 0.674106336 |
|  | e6 | TOPK | 0.701508157 | 0.535193948 |
|  | e7 | ZAK | 0.842104862 |  |
|  | e8 | PDK4 | 1.10144182 |  |
|  | e9 | CHUK | 0.852075221 |  |
|  | e10 | TPK1 | 0.525508259 | 0.688400324 |
|  | e11 | C14ORF20 | 0.707144754 | 0.686361139 |
|  | f2 | PIM3 | 0.793490827 |  |
|  | f3 | LMTK3 | 0.97562614 |  |
|  | f4 | CHEK2 | 0.763219422 |  |
|  | f5 | JAK1 | 0.748863953 | 0.721269164 |
|  | f6 | EIF2AK3 | 0.93056059 |  |
|  | f7 | PIK3C3 | 0.545329093 | 0.835377514 |
|  | f8 | ROCK2 | 0.669986774 | 0.815463175 |
|  | f9 | PIK3C2B | 0.666013353 | 0.807160127 |
|  | f10 | SGK | 0.768050007 |  |
|  | f11 | CLK3 | 1.213134863 |  |
|  | g2 | PXK | 0.493579238 | 0.827148328 |
|  | g3 | HUNK | 0.995634589 |  |
|  | g4 | MAP4K1 | 0.957824714 |  |
|  | g5 | PCTK2 | 0.769224543 |  |
|  | g6 | INSR | 0.711130699 | 1.585183387 |
|  | g7 | HIPK4 | 1.103096867 |  |
|  | g8 | SIK2 | 0.451157439 | 1.200312083 |
|  | g9 | MET | 1.554059648 |  |
|  | g10 | FYN | 0.910300915 |  |
|  | g11 | DKFZP434C131 | 0.927174822 |  |
|  | h2 | SAST | 0.952293944 |  |
|  | h3 | NEK2 | 0.835616284 |  |
|  | h4 | MAPKAPK5 | 1.037504953 |  |
|  | h5 | NRK | 0.907735025 |  |
|  | h6 | PFKFB2 | 0.871218889 |  |
|  | h7 | MARK4 | 0.934822144 |  |
|  | h8 | EPHB1 | 0.49825247 | 1.400963274 |
|  | h9 | MAGI-3 | 1.006507439 |  |
|  | h10 | SSTK | 0.996576733 |  |
|  | h11 | IKBKG | 0.930740537 |  |
| p9 | a2 | N4BP2 | 2.07346722 | 2.598723373 |
|  | a3 | NEK1 | 2.95757203 | 1.541215994 |
|  | a4 | XYLB | 1.578518631 |  |
|  | a5 | RP6-213H19.1 | 2.419923813 | 2.076701421 |
|  | a6 | SMG1 | 1.738032111 | 2.296396086 |
|  | a7 | IHPK2 | 1.970705552 | 1.912912654 |
|  | a8 | ILK | 1.03226679 |  |
|  | a9 | PSKH2 | 1.720963297 | 0.778152372 |
|  | a10 | NRBP | 1.75236685 | 0.760260158 |
|  | a11 | ULK1 | 1.780269731 | 1.130330178 |
|  | b2 | PRKCA | 2.770961655 | 2.282882574 |
|  | b3 | CDKL2 | 2.189081445 | 2.00793105 |
|  | b4 | FES | 1.99622035 | 3.849305607 |
|  | b5 | PRKAB1 | 1.60995022 | 0.73922274 |
|  | b6 | HAK | 1.560856154 | 1.164940654 |
|  | b7 | CDK2 | 2.318231399 | 1.575725364 |
|  | b8 | MAP2K2 | 1.653016828 | 1.484066286 |
|  | b9 | MAPK10 | 1.734385025 | 1.219651777 |
|  | b10 | CALM2 | 1.901465261 | 1.216589376 |
|  | b11 | KHK | 2.481762351 | 6.794888023 |
|  | c2 | GUK1 | 1.275245154 |  |
|  | c3 | CKM | 0.98527093 |  |
|  | c4 | MAP4K3 | 0.852563174 |  |
|  | c5 | PRKDC | 0.89261854 |  |
|  | c6 | PRPF4B | 0.49834028 | 2.774399354 |
|  | c7 | RIOK3 | 0.923409941 |  |
|  | c8 | KIAA1765 | 0.695105673 |  |
|  | c9 | EXOSC10 | 0.760069034 |  |
|  | c10 | PGK1 | 0.971061927 |  |
|  | c11 | AK3L1 | 0.912467194 |  |
|  | d2 | PLK3 | 0.813853939 |  |
|  | d3 | EFNB3 | 1.257174685 |  |
|  | d4 | EPHB3 | 0.71175479 |  |
|  | d5 | PRKCB1 | 0.717763075 |  |
|  | d6 | DGKD | 0.633352839 |  |
|  | d7 | EPHB4 | 1.071882329 |  |
|  | d8 | MGC45428 | 1.236770831 |  |
|  | d9 | CHKB | 1.01985855 |  |
|  | d10 | PRKAR2B | 0.676112393 |  |
|  | d11 | PCK2 | 1.340167784 |  |
|  | e2 | AMHR2 | 0.924212785 |  |
|  | e3 | DUSP21 | 0.657447839 |  |
|  | e4 | CDKN2B | 0.86553915 |  |
|  | e5 | DLG1 | 0.993545859 |  |
|  | e6 | C9ORF96 | 0.910712037 |  |
|  | e7 | MAP2K1 | 0.885015116 |  |
|  | e8 | GALK2 | 0.791708954 |  |
|  | e9 | NTRK1 | 0.735247247 |  |
|  | e10 | NME4 | 0.559533172 | 1.45286632 |
|  | e11 | BRAF | 0.681800797 |  |
|  | f2 | PASK | 1.055015715 |  |
|  | f3 | HUS1 | 1.00041698 |  |
|  | f4 | TLK1 | 0.964439165 |  |
|  | f5 | PFKM | 0.780333503 |  |
|  | f6 | PRKAA2 | 1.15986498 |  |
|  | f7 | ITPKB | 0.876170696 |  |
|  | f8 | MPP3 | 0.813829071 |  |
|  | f9 | CDK3 | 1.074803141 |  |
|  | f10 | PDPK1 | 0.778588004 |  |
|  | f11 | C10ORF89 | 0.914060526 |  |
|  | g2 | EPHA4 | 1.696128215 | 0.703767064 |
|  | g3 | DCAMKL1 | 1.315577629 |  |
|  | g4 | GK2 | 0.808356111 |  |
|  | g5 | KUB3 | 0.703075342 |  |
|  | g6 | TRPM7 | 0.787049343 |  |
|  | g7 | MYLK | 0.572680832 | 0.833516724 |
|  | g8 | GUCY2F | 0.820181405 |  |
|  | g9 | GSK3B | 0.832746531 |  |
|  | g10 | MST1R | 0.893916593 |  |
|  | g11 | AK2 | 0.913505665 |  |
|  | h2 | NEK7 | 1.555676975 | 4.489314352 |
|  | h3 | PRKWNK1 | 0.741538567 |  |
|  | h4 | TSKS | 0.864227111 |  |
|  | h5 | STK19 | 0.826728451 |  |
|  | h6 | MAPK12 | 1.002516533 |  |
|  | h7 | GALK1 | 1.038281554 |  |
|  | h8 | IPMK | 1.054559711 |  |
|  | h9 | PANK4 | 0.558478447 | 0.935527182 |
|  | h10 | PRKACG | 0.971836856 |  |
|  | h11 | FLT1 | 0.924366812 |  |
